# Timing of therapies for Down syndrome: the sooner, the better

**DOI:** 10.3389/fnbeh.2015.00265

**Published:** 2015-10-06

**Authors:** Fiorenza Stagni, Andrea Giacomini, Sandra Guidi, Elisabetta Ciani, Renata Bartesaghi

**Affiliations:** Department of Biomedical and Neuromotor Sciences, University of BolognaBologna, Italy

**Keywords:** Down syndrome, intellectual disability, mouse models, adult therapies, perinatal therapies

## Abstract

Intellectual disability (ID) is the unavoidable hallmark of Down syndrome (DS), with a heavy impact on public health. Accumulating evidence shows that DS is characterized by numerous neurodevelopmental alterations among which the reduction of neurogenesis, dendritic hypotrophy and connectivity alterations appear to play a particularly prominent role. Although the mechanisms whereby gene triplication impairs brain development in DS have not been fully clarified, it is theoretically possible to correct trisomy-dependent defects with targeted pharmacotherapies. This review summarizes what we know about the effects of pharmacotherapies during different life stages in mouse models of DS. Since brain alterations in DS start to be present prenatally, the prenatal period represents an optimum window of opportunity for therapeutic interventions. Importantly, recent studies clearly show that treatment during the prenatal period can rescue overall brain development and behavior and that this effect outlasts treatment cessation. Although late therapies are unlikely to exert drastic changes in the brain, they may have an impact on the hippocampus, a brain region where neurogenesis continues throughout life. Indeed, treatment at adult life stages improves or even rescues hippocampal neurogenesis and connectivity and hippocampal-dependent learning and memory, although the duration of these effects still remains, in the majority of cases, a matter of investigation. The exciting discovery that trisomy-linked brain abnormalities can be prevented with early interventions gives us reason to believe that treatments during pregnancy may rescue brain development in fetuses with DS. For this reason we deem it extremely important to expedite the discovery of additional therapies practicable in humans in order to identify the best treatment/s in terms of efficacy and paucity of side effects. Prompt achievement of this goal is the big challenge for the scientific community of researchers interested in DS.

Intellectual disability (ID) is the most serious problem of Down syndrome (DS), with a heavy impact on families and society. Intense efforts of scientists worldwide are currently trying to discover interventions that improve or even rescue ID in DS. The results summarized below suggest that therapy for DS may be possible and that appropriately timed therapies may have a large impact on ID. This achievement would give children with DS the opportunity of a normal and autonomous life, alleviate the psychological burden on their families and solve a public health problem. This review summarizes therapies attempted in mouse models of DS, focusing in particular on early interventions.

## DS is characterized by brain defects that can be traced back to fetal life stages

IQ in people with DS usually falls in the moderately to severely retarded range (IQ = 25–55) and mental age is rarely over 8 years (See Rachidi and Lopes, 2008; Dierssen, 2012). The IQ in DS is not constant during life but decreases with age and an early deceleration occurs between the age of 6 months and 2 years, with a further decline in adolescents. Children with DS exhibit incomplete and delayed acquisition of motor, linguistic, cognitive, and adaptive functions, compared with developing children of the same mental age. The brain of a child with DS develops differently from a normal brain and attains a form that is reduced in size and altered in shape. Widespread neurogenesis impairment has been documented in fetuses with DS (Contestabile et al., 2007; Guidi et al., 2008, 2011) and in mouse models of DS (Chakrabarti et al., 2007; Bianchi et al., 2010a,b; Trazzi et al., 2011) during critical brain developmental stages and is one of the major determinants of ID in DS. Proliferation impairment is worsened by a reduction in the acquisition of a neuronal phenotype and a relative increase in astrogliogenesis. In contrast, there is an increase in the production of inhibitory neurons that causes an excitation/inhibition imbalance (Chakrabarti et al., 2010). In addition to neurogenesis impairment, the DS brain is characterized by dendritic hypotrophy, spine density reduction, and alterations in spine shape (Takashima et al., 1989; Becker et al., 1991; Belichenko et al., 2004; Benavides-Piccione et al., 2004; Guidi et al., 2013) and widespread alterations of various transmitter and receptor systems (see Bartesaghi et al., 2011). These defects, which imply altered network formation and functioning, are also important determinants of ID in DS.

## The Ts65Dn mouse: a widely used model for studying DS

Various mouse models have been created that are trisomic for different sets of genes of Hsa21. Animal models do not reproduce the human disease with all its complexities but rather model specific aspects of the disease and no perfect model of DS exists. The Ts65Dn mouse is the most studied and best characterized model of DS. It bears segmental trisomy for a distal region of Mmu16 that contains approximately 55% of Hsa21 conserved genes (Davisson et al., 1990). This model is additionally trisomic for approximately 50 genes that are non-hortologous to Hsa21 (Rueda et al., 2012). During the past 20 years, numerous studies have demonstrated common features between Ts65Dn and humans, and the Ts65Dn mouse is, at the moment, the only model of DS used in pre-clinical studies to develop therapies for DS (Gardiner, 2015). However, there are some aspects that make this model limited. (1) The Ts65Dn mouse lacks numerous Hsa21 orthologous genes and has some Mmu17 genes that are non-trisomic in humans. These genes may confound results of therapeutic interventions. (2) Since males are sterile, mice are generated from Ts65Dn dams. The trisomic condition of mothers could cause developmental problems of the pups independently from trisomy. Along the same line of reasoning, embryonic treatments may have beneficial effects on trisomic pups that are secondary to the beneficial effects on the trisomic dams. Due to these limitations, treatment on Ts65Dn mice may have an unpredictable clinical outcome. Nevertheless, the Ts65Dn mouse has allowed scientists to discover treatments that may also be beneficial in individuals with DS.

## Brain functions in DS can be pharmacologically improved

The mechanisms whereby gene triplication leads to brain developmental alteration and, hence, ID remain to be elucidated. Among the triplicated genes *DYRK1A, SIM2, DSCAM, GIRK2, Olig1*, and *Olig2, SYNJ1*, and *APP* are thought to be heavily involved in the DS neurological phenotype. Moreover, *APP* triplication appears to be a key factor that favors the almost unavoidable development of Alzheimer's disease in adults with DS. Ideally, identification of the molecular mechanisms underlying brain abnormalities in DS will provide a rational basis from which to devise therapies that, by targeting specific cellular pathway/s, may correct the developmental defects of the DS brain. Although the molecular mechanisms that disrupt brain development in DS have not been fully clarified so far, various therapies have been attempted during the past few years in the Ts65Dn mouse model showing that it is possible to pharmacologically improve cognitive performance and different aspects of the DS brain phenotype (Tables 1, 2).

**Table 1:** **Therapies administered at adult life stages in the Ts65Dn mouse model of DS**.

**Phenotype**	**Treatment**	**Mechanism**	**Age (M)**	**Treatment duration**	**Outcome**	**Long-term effect**	**References**
L/M (MWM)	Donepezil (Class A)	AChE inhibitor	4	7 w	**Failed**	NA	Rueda et al., 2008a
L/M (SA)	Physostigmine (Class A)	AChE inhibitor	4 10 16	Acute Acute Acute	Rescued **Failed** **Failed**	NA NA NA	Chang and Gold, 2008
Olfactory learning	Galantamine (Class A)	AChE inhibitor	3–6	Acute	Rescued	NA	de Souza et al., 2011
L/M (NOR, TM)	Pentylentetrazole (Class A)	Antagonist of GABA_A_ R	3–4	17 d	Rescued	**Yes (at 2 m)**	Fernandez et al., 2007
L/M (MWM)	Pentylentetrazole (Class A)	Antagonist of GABA_A_ R	4	7 w	Rescued	NA	Rueda et al., 2008a
L/M (NOR)	Pentylentetrazole (Class A)	Antagonist of GABA_A_ R	2–3	2 w	Rescued	**Yes (at 8 d)**	Colas et al., 2013
L/M (NOR)	Pentylentetrazole (Class A)	Antagonist of GABA_A_ R	12–15	2 w	Rescued	**Yes (at 8 d)**	Colas et al., 2013
L/M (MWM)	RO4938581 (Class A)	GABA_A_ α5 negative allosteric modulator	3–4	6 w	Rescued	NA	Martínez-Cué et al., 2013
L/M (NOR, MWM, CFC)	CGP55845 (Class A)	Antagonist of GABA_B_ R	2–3	3 w	Rescued	NA	Kleschevnikov et al., 2012
L/M (MWM, CFC)	Ethosuximide (Class A)	Inhibits KCNJ6/GIRK2 channel, a GABA_B_–coupled ion channel	4.5–5	10 w	**Failed**	NA	Vidal et al., 2012
L/M (MWM, CFC)	Gabapentin (Class A)	Modulator of GABA synthesis	4.5–5	10 w	**Failed**	NA	Vidal et al., 2012
L/M (CFC, nesting behavior)	L-DOPS (Class A)	NA pro-drug	6	Acute	Rescued	**No (at 2 w)**	Salehi et al., 2009
L/M (NOR, CFC, TM)	Xamoterol (Class A)	β1 receptor agonist	9–12	Acute	Rescued	NA	Faizi et al., 2011
L/M (NOR, SA)	Clozapine-N-oxide (agonist of hM3Dq, administered via adeno virus into Locus Coeruleus) (Class A)	DREADD design in order to stimulate NA neurons of Locus Coeruleus	14	Acute	Rescued	NA	Fortress et al., 2015
L/M (SA)	L-DOPS (Class A)	NA pro-drug	11	2 w	Rescued	NA	Fortress et al., 2015
L/M (CFC)	Memantine (Class A)	Antagonist of NMDA R	4–7	Acute	Rescued	NA	Costa et al., 2008; Ahmed et al., 2015
L/M (WRAM, NOR)	Memantine (Class A)	Antagonist of NMDA R	4	6 m	Improved	**No (at 1 w)**	Lockrow et al., 2011
L/M (MWM)	Memantine (Class A)	Antagonist of NMDA R	9	8–9 w	Rescued	NA	Rueda et al., 2010
L/M (YM)	RO25-6981 (Class A)	Antagonist of NMDA R (GluN2B)	3–6	Acute	**Failed**	NA	Hanson et al., 2013
L/M (YM, BM)	RO25-6981 (Class A)	Antagonist of NMDA R (GluN2B)	3–6	2 w	**Failed**	NA	Hanson et al., 2013
L/M (NOR, YM)	Fluoxetine (Class A)	Inhibits serotonine reuptake	> 2 m	8 w	Rescued	NA	Begenisic et al., 2014
L/M (MWM)	Fluoxetine (Class A)	Inhibits serotonine reuptake	5–7	4 w	**Failed**	NA	Heinen et al., 2012
L/M (YM, NPR, NOR)	JZL184 (Class A)	Inhibitor of monoacylglycerol lipase that increases levels of 2-arachidonoylglycerol	11	4 w	**Failed** (YM, NPR) Rescued (NOR)	NA	Lysenko et al., 2014
L/M (MWM)	NAPVSIPQ+SALLRSIPA (fragments of ADNP and ADNF) (Class B)	Neuroprotection against oxidative stress	10	9 d	Rescued	**No (at 10 d)**	Incerti et al., 2011
L/M (MWM)	Peptide six (fragment of CNTF) (Class B)	Neurotrophic factor	11–15	30 d	Improved	NA	Blanchard et al., 2011
L/M (TM)	Estrogen (Class B)	Protects basal forebrain cholinergic neurons	11–15	2 m	Improved	NA	Granholm et al., 2002
L/M (MWM, PM)	Melatonin (Class B)	Free radical scavenger	5–6	5 m	Improved	NA	Corrales et al., 2013
L/M (WRAM)	Vitamin E (Class B)	Antioxidant	4	4–6 m	Improved	NA	Lockrow et al., 2009
L/M (MWM)	Piracetam (Class B)	Nootropic	1.3	4 w	**Failed**	NA	Moran et al., 2002
L/M (MWM)	SGS-111 (Class B)	Analog of Piracetam. Nootropic	4–6	6 w	**Failed**	NA	Rueda et al., 2008b
L/M (WRAM)	Minocycline (Class B)	Anti-inflammatory	7	3 m	Improved	NA	Hunter et al., 2004
L/M (MWM, NOR, CFC)	Lithium (Class C)	Mood stabilizer. Interferes with GSK3β signaling	5–6	4 w	Rescued	NA	Contestabile et al., 2013
L/M (MWM)	DAPT (Class D)	Gamma-secretase inhibitor	4	Acute	Rescued	NA	Netzer et al., 2010
L/M (MWM, NOR)	Epigallocatechin-3-gallate (EGCG) (Class D)	Inhibitor of DYRK1A kinase	3	1 m	Rescued	NA	De la Torre et al., 2014
LTP	Pentylentetrazole (Class A)	GABA_A_ R antagonist	3–4	17 d	Rescued	**Yes (at 2 m)**	Fernandez et al., 2007
LTP	RO4938581 (Class A)	GABA_A_ α5 negative allosteric modulator	3–4	6 w	Rescued	NA	Martínez-Cué et al., 2013
LTP	CGP55845 (Class A)	Antagonist of GABA_B_ R	2–3	3 w	Rescued	NA	Kleschevnikov et al., 2012
LTP	Picrotoxin (Class A)	Antagonist of GABA_A_R	3–4	Acute	Rescued	Acute (slices)	Kleschevnikov et al., 2004
LTP	Picrotoxin (Class A)	Antagonist of GABA_A_R	4–6	Acute	Rescued	Acute (slices)	Costa and Grybko, 2005
LTP	RO25-6981 (Class A)	Antagonist of NMDA R (GluN2B)	3–6	2 w	Rescued	**Yes (at 2–4.5 w)**	Hanson et al., 2013
LTP	Fluoxetine (Class A)	Inhibits serotonine reuptake	> 2	8 w	Rescued	NA	Begenisic et al., 2014
LTP	JZL184 (Class A)	Inhibitor of monoacylglycerol lipase/Endocann System	11	4 w	Improved	NA	Lysenko et al., 2014
LTP	Melatonin (Class B)	Free radical scavenger	6–6.5	5–5.5 m	Rescued	NA	Corrales et al., 2014
LTP	Lithium (Class C)	Mood stabilizer. Interferes with GSK3β signaling	5–6	4 w	Rescued	NA	Contestabile et al., 2013
LTP	Epigallocatechin-3-gallate (EGCG) (Class D)	Inhibitor of DYRK1A kinase	2–5	Acute	Rescued	Acute (slices)	Xie et al., 2008
Neurogenesis (DG)	RO4938581 (Class A)	GABA_A_ α5 negative allosteric modulator	3–4	6 w	Rescued	NA	Martínez-Cué et al., 2013
Neurogenesis (DG)	Farmoterol (Class A)	β2 Receptor agonist	5–6	15 d	**Failed**	NA	Dang et al., 2014
Neurogenesis (DG)	Fluoxetine (Class A)	Inhibits serotonin reuptake	2–5	24 d	Rescued	NA	Clark et al., 2006
Neurogenesis (DG)	Peptide six (fragment of CNTF) (Class B)	Neurotrophic factor	11–15	30 d	Rescued	NA	Blanchard et al., 2011
Neurogenesis (DG)	Melatonin (Class B)	Free radical scavenger	6–6.5	5–5.5 m	Rescued	NA	Corrales et al., 2014
Neurogenesis (DG)	Lithium (Class C)	Mood stabilizer. Interferes with GSK3β signaling	5–6	4 w	Rescued	NA	Contestabile et al., 2013
Neurogenesis (SVZ)	Lithium (Class C)	Mood stabilizer. Interferes with GSK3β signaling	12	1 m	Rescued	NA	Bianchi et al., 2010a
Neurogenesis (DG)	P7C3 (Class E)	Proneurogenic drug	1–2.5	3 m	Improved	NA	Latchney et al., 2015
Dendritic hypotrophy	Farmoterol (Class A)	β2 Receptor agonist	5–6	15 d	Rescued	NA	Dang et al., 2014
Connectivity	Peptide six (fragment of CNTF) (Class B)	Neurotrophic factor	11–15	30 d	Rescued	NA	Blanchard et al., 2011
Neurodegeneration	Estrogen (Class B)	Protects basal forebrain cholinergic neurons	11–15	2 m	Rescued	NA	Granholm et al., 2002
Neurodegeneration	Estrogen (Class B)	Protects basal forebrain cholinergic neurons	9–15	2 m	Rescued	NA	Granholm et al., 2003
Neurodegeneration	Minocyclin (Class B)	Anti-inflammatory	7	3 m	Prevented	NA	Hunter et al., 2004
Neurodegeneration	Vitamin E (Class B)	Antioxidant	4	4–6 m	Prevented	NA	Lockrow et al., 2009

*The classes reported in the column “Treatment” correspond to those summarized in the Section “Numerous Therapies Have Been Attempted in Order to Improve the Phenotype of the Trisomic Brain.” The outcome “Rescued” means that in treated Ts65Dn mice the examined phenotype became similar to that of untreated euploid mice. The outcome “Improved” means that in Ts65Dn mice treatment ameliorated but did not rescue the examined phenotype. The text in bold in the column “Outcome” highlights treatments that were ineffective (Failed) and the text in bold in the column “Long-term effect” highlights treatments that either had (Yes) or did no had (No) a long-term effect. ADNF, Activity Dependent Neurotrophic Factor; ADNP, Activity Dependent Neuroprotective Protein; BM, Barnes Maze; CFC, Contextual Fear Conditioning; CNTF, Ciliary Neurotrophic Factor; d, day; DG, dentate gyrus; m, month; MWM, Morris Water Maze; NA, not available; NOR, Novel Object Recognition; NPR, Novel Place Recognition; PM, Plus Maze; SA, Spontaneous Alternation Task; SVZ, subventricular zone; TM, T Maze; w, week; WRAM, Water Radial Arm Maze; YM, Y Maze*.

**Table 2:** **Therapies administered at neonatal and embryonic life stages in the Ts65Dn mouse model of DS**.

**Phenotype**	**Treatment**	**Mechanism**	**Age**	**Duration**	**Outcome**	**Long-term effect**	**References**
**NEONATAL TREATMENT**							
L/M (CFC)	Fluoxetine (Class A)	Inhibits serotonine reuptake	P3	13 d	Rescued	**Yes (at 1 m)**	Bianchi et al., 2010a
L/M (MWM, NOR, PA)	Fluoxetine (Class A)	Inhibits serotonine reuptake	P3	13 d	Rescued	**Yes (at 3 m)**	Stagni et al., 2015
L/M (YM)	SAG (Class C)	Synthetic activator of Sonic hedgehog pathway	P0	1 injection	**Failed**	**Yes (at 4 m)**	Das et al., 2013
L/M (MWM)	SAG (Class C)	Synthetic activator of Sonic hedgehog pathway	P0	1 injection	Rescued	**Yes (at 4 m)**	Das et al., 2013
LTP (CA1)	SAG (Class C)	Synthetic activator of Sonic hedgehog pathway	P0	1 injection	Rescued	**Yes (at 4 m)**	Das et al., 2013
Cerebellar-functional deficits	SAG (Class C)	Synthetic activator of Sonic hedgehog pathway	P0	1 injection	**Failed**	**Yes (at 4 m)**	Gutierrez-Castellanos et al., 2013
Neurogenesis (DG and SVZ)	Fluoxetine (Class A)	Inhibits serotonin reuptake	P3	13 d	Rescued	**Yes (at 1 m)**	Bianchi et al., 2010a
Neurogenesis (DG and SVZ)	Fluoxetine (Class A)	Inhibits serotonin reuptake	P3	13 d	Rescued	**Yes (at 3 m)**	Stagni et al., 2015
Neurogenesis (DG)	SAG (Class C)	Synthetic activator of Sonic hedgehog pathway	P0	1 injection	**Failed**	**Yes (at 6 d)**	Das et al., 2013
Neurogenesis (Cerebellar granule cells)	SAG (Class C)	Synthetic activator of Sonic hedgehog pathway	P0	1 injection	Rescued	NA	Roper et al., 2006
Neurogenesis (DG and SVZ)	Epigallocatechin-3-gallate (Class D)	Inhibits DYRK1A kinase	P3	13 d	Rescued	NA	Stagni et al., 2014
Cellularity (DG granule cells)	Fluoxetine (Class A)	Inhibits serotonin reuptake	P3	13 d	Rescued	**Yes (at 1 m)**	Bianchi et al., 2010a
Cellularity (DG granule cells)	Fluoxetine (Class A)	Inhibits serotonin reuptake	P3	13 d	Rescued	**Yes (at 3 m)**	Stagni et al., 2015
Cellularity (Cerebellar granule cells)	SAG (Class C)	Synthetic activator of Sonic hedgehog pathway	P0	1 injection	Rescued	**Yes (at 4 m)**	Das et al., 2013
Cellularity (DG granule cells)	Epigallocatechin-3-gallate (Class D)	Inhibits DYRK1A kinase	P3	13 d	Rescued	NA	Stagni et al., 2014
Dendritic hypotrophy	Fluoxetine (Class A)	Inhibits serotonin reuptake	P3	13 d	Rescued	**Yes (at 1 m)**	Guidi et al., 2013
Dendritic hypotrophy	Fluoxetine (Class A)	Inhibits serotonin reuptake	P3	13 d	Rescued	**Yes (at 3 m)**	Stagni et al., 2015
Connectivity	Fluoxetine (Class A)	Inhibits serotonin reuptake	P3	13 d	Rescued	**Yes (at 1 m)**	Stagni et al., 2013
Connectivity	Fluoxetine (Class A)	Inhibits serotonin reuptake	P3	13 d	Rescued	**Yes (at 3 m)**	Stagni et al., 2015
**PRENATAL TREATMENT**							
L/M (RAWM)	Choline supplement (Class A)	Precursor of acetylcholine	Dams	E + 21 d	Improved	**Yes (at 13–17 m)**	Velazquez et al., 2013
Visual attention tasks	Choline supplement (Class A)	Precursor of acetylcholine	Dams	E + 21 d	Improved	**Yes (at 6–12 m)**	Moon et al., 2010
L/M (RAWM)	Choline supplement (Class A)	Precursor of acetylcholine	Dams	E + 21 d	Improved	**Yes (at 13–17 m)**	Ash et al., 2014
L/M (CFC)	Fluoxetine (Class A)	Inhibits serotonin reuptake	E10	Up to E20/21	Rescued	**Yes (at 1.5 m)**	Guidi et al., 2014
Motor and sensory milestones	NAPVSIPQ+SALLRSIPA (Class B)	Active fragments of ADNP and ADNF	E8	Up to E12	Rescued	**Yes (at P5–P20)**	Toso et al., 2008
L/M (MWM)	NAPVSIPQ+SALLRSIPA (Class B)	Active fragments of ADNP and ADNF	E8	Up to E12	Rescued	**Yes (at 8–10 m)**	Incerti et al., 2012
L/M (MWM)	Vitamin E (Class B)	Antioxidant	Dams	E+12 w	Improved	NA	Shichiri et al., 2011
Motor and sensory milestones L/M (MWM, PA)	SGS-111 (Class B)	Analog of Piracetam; Nootropic	Dams	E+5 m	**Failed**	NA	Rueda et al., 2008b
Neurogenesis (DG)	Choline supplement (Class A)	Precursor of acetylcholine	Dams	E + 21 d	Improved	**Yes (at 13–17 m)**	Velazquez et al., 2013
Neurogenesis (all brain regions)	Fluoxetine (Class A)	Inhibits serotonin reuptake	E10	Up to E20/21	Rescued	**Yes (at 1.5 m)**	Guidi et al., 2014
Cellularity (all brain regions)	Fluoxetine (Class A)	Inhibits serotonin reuptake	E10	Up to E20/21	Rescued	**Yes (at 1.5 m)**	Guidi et al., 2014
Cellularity (DG granule cells)	A-tochopherol (Class B)	Antioxidant	Dams	E+12 w	Rescued	NA	Shichiri et al., 2011
Dendritic hypotrophy	Fluoxetine (Class A)	Inhibits serotonin reuptake	E10	Up to E20/21	Rescued	**Yes (at 1.5 m)**	Guidi et al., 2014
Connectivity	Fluoxetine (Class A)	Inhibits serotonin reuptake	E10	Up to E20/21	Rescued	**Yes (at 1.5 m)**	Guidi et al., 2014
Neurodegeneration	Choline supplement (Class A)	Precursor of acetylcholine	Dams	E + 21 d	Improved	**Yes (at 13–17 m)**	Ash et al., 2014
Neurodegeneration	Choline supplement (Class A)	Precursor of acetylcholine	Dams	E + 21 d	Improved	**Yes (at 4.3–7.5 m)**	Kelley et al., 2014

*The classes reported in the column “Treatment” correspond to those summarized in the Section “Numerous Therapies Have Been Attempted in Order to Improve the Phenotype of the Trisomic Brain.” The outcome “Rescued” means that in treated Ts65Dn mice the examined phenotype became similar to that of untreated euploid mice. The outcome “Improved” means that in Ts65Dn mice treatment ameliorated but did not rescue the examined phenotype. The text in bold in the column “Outcome” highlights treatments that were ineffective (Failed) and the text in bold in the column “Long-term effect” highlights treatments that either had (Yes) or did no had (No) a long-term effect. CFC, Contextual Fear Conditioning; d, day; DG, dentate gyrus; E, embryonic; m, month; MWM, Morris Water Maze; NA, Not Available; NOR, Novel Object Recognition; PM, Plus Maze; TM, T Maze; RAWM, Radial Arm Water Maze; SVZ, subventricular zone; w, week; YM, Y Maze*.

## The number of pre-clinical studies for DS has progressively increased during the past few years

**Figure 1:**
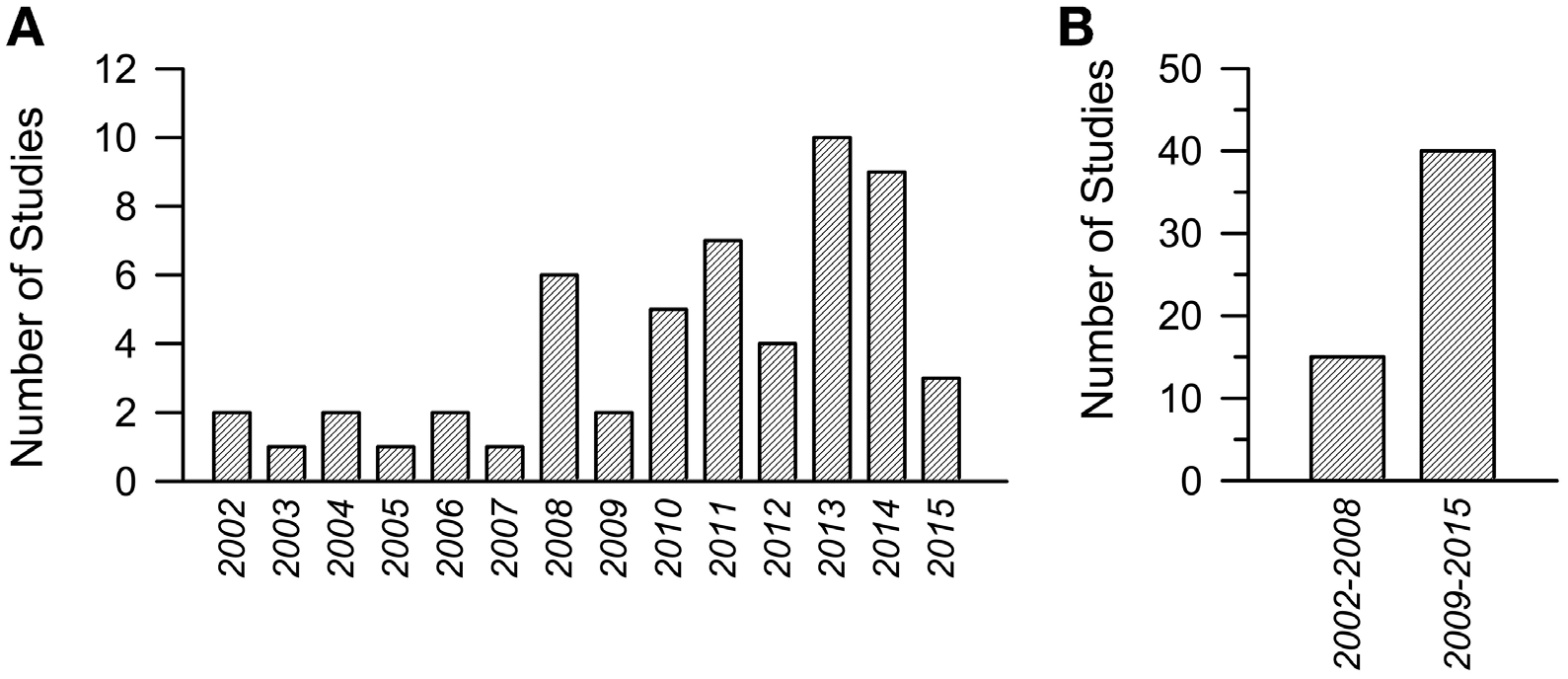
**Number of studies focused on pharmacotherapies in Down syndrome mouse models in the period 2002–2015**. **(A)** Number or studies per year. **(B)** Cumulative number of studies in the period 2002–2008 and 2009–2015.

During the past 14 years the number of studies focusing on pharmacotherapies for DS has grown almost exponentially. The results of a Medline research [a group of keywords was: “Down syndrome AND mouse model AND (therapy OR treatment OR restoration OR rescue OR improvement)”; a second group of keywords was: “Down syndrome AND mouse model AND LTP”] are summarized in Figure 1. Figure 1A summarizes the number of articles published since 2002 up to the beginning of current year. While in the period 2002–2008 the overall number of articles was 15 (Figure 1B), with a mean number of two articles per year, in the period 2009–2015 the overall number of articles was 40 (Figure 1B), with a mean number of six articles per year. These figures are quite encouraging because they show that the relatively small community of researchers interested in DS is making increasing efforts to find treatment for DS. This gives us hope that this intense commitment will produce good results in a near future.

## Numerous therapies have been attempted in order to improve the phenotype of the trisomic brain

A number of therapies have been tested so far in mouse models of DS in order to improve the DS-linked brain phenotype. Since most of these therapies have been tested in the Ts65Dn mouse, the most popular model of DS, we will focus here mainly on therapies tested in this model. These therapies, which have been selected according to different rationales, can be variously classified, according to the chosen common denominators. Here we have grouped the attempted therapies into five major classes, named A–E (also reported in Tables 1, 2). (A) Therapies targeted to transmitter systems. (i) Therapies enhancing cholinergic trasmission in order to counteract age-related damage of the cholinergic systems; (ii) Therapies antagonizing GABAergic transmission, in order to reduce excessive inhibition; (iii) Therapies enhancing noradrenergic transmission, in order to compensate for dysfunctions of noradrenergic afferents to the hippocampus; (iv) Therapies targeted to the glutamate NMDA receptor, in order to restore its function; (v) Therapies targeted to the serotonergic system, in order to enhance defective serotonergic signaling; (vi) Therapies targeted to the endocannabinoid system, in order to increase its activity. (B) Therapies employing neuroprotective agents, antioxidants, and free radical scavengers, in order to reduce neurodegeneration, a typical feature of the DS brain. (C) Therapies targeted to perturbed signaling pathways. (D) Therapies to normalize the expression of proteins coded by triplicated genes. (E) Therapies that are known to have a proneurogenic effect.

**Figure 2:**
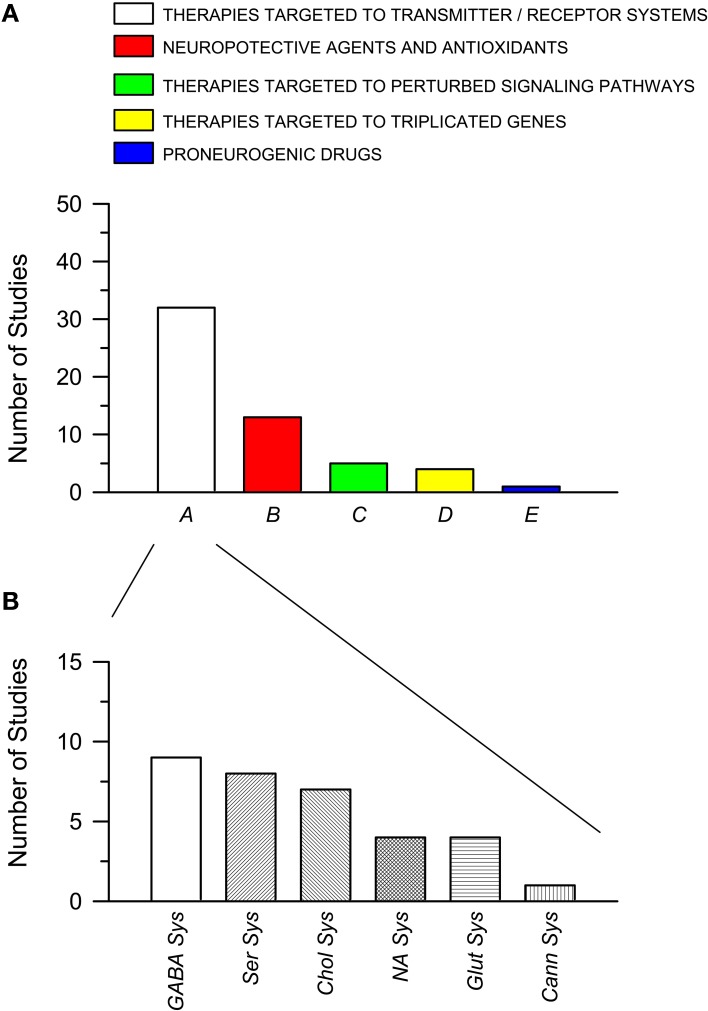
**Pharmacotherapies used in the Ts65Dn mouse models of Down syndrome grouped per class**. **(A)** Pharmacotherapies grouped into five classes **(A–E)**, as explained in the text. The histogram shows the number of published studies for each class. **(B)** Number of studies for each subclass of pharmacotherapies belonging to class A (see text for explanation). Abbreviations: Cann Sys, cannabinoid sysem; Chol Sys, cholinegic system; GABA Sys, GABAergic system; Glut Sys, glutamatergic system; NA Sys, noradrenergic system; Ser Sys, serotonergic system.

The total number of studies for each of these five classes is shown in Figure 2A. It is evident that more than one half of the studies (32 out of a total of 55) that have attempted to rescue DS brain phenotypes have used drugs that act on transmitter systems. Many transmitter systems are altered in DS and by correcting altered synaptic function it may be possible to reinstate signal transfer, on one hand, and activity-dependent cellular functions, on the other. Most of the studies belonging to class A focus on the GABAergic system (Figure 2B). The rationale is that since an excessive inhibition characterizes the trisomic brain, it may be possible to normalize its function by reducing inhibition. The second most numerous group of therapies belongs to class B. This class may expand if we shift therapies targeted to the cholinergic system from class A to class B. The rationale for the wide use of neuroprotective agents or antioxidants depends on the fact that the trisomic brain undergoes neurodegeneration and develops an Alzheimer's-like pathology with age. Thus, neuroprotective agents may prevent or delay neurodegeneration. Of course, the classification criteria are not entirely flaw-free and categories may be overlapping. For instance, therapies acting on the cholinergic system may belong to class A of this review as well as to class B. The outcomes of therapies of the different classes can be found in Tables 1, 2. Note that this review reports results of pharmacological interventions that have examined one or more of these phenotypic features: learning and memory, LTP, neurogenesis/cellularity, dendritic pattern, and neurodegeneration. Therapies based on non-pharmacological approaches have not been included.

## Trisomy-linked brain phenotypes can be rescued by different therapies

**Figure 3:**
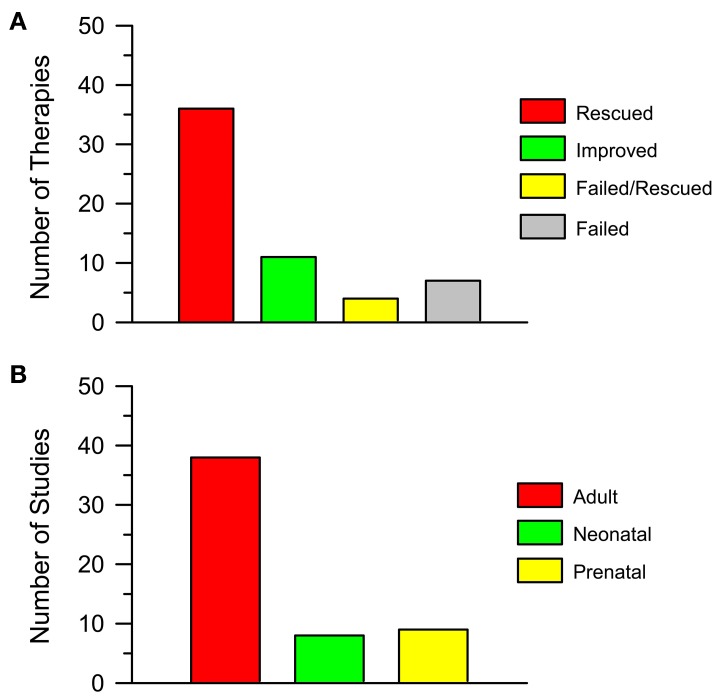
**Pharmacotherapies in the Ts65Dn mouse model of Down syndrome. (A)** The histogram shows the number of attempted pharmacotherapies in the Ts65Dn mouse model of DS that rescued (Rescued), improved (Improved), partially rescued (Failed/Rescued) the examined phenotype/s or had no effect (Failed). Data derive from Tables 1, 2. **(B)** The histogram shows the number of studies in which pharmacotherapies were administered at adult life stages, in the neonatal period and in the prenatal (in some instances plus post-natal) period. Data derive from Tables 1, 2.

By looking at Tables 1, 2 it appears that a variety of different agents, that act on different targets, can rescue one or more of the DS brain phenotypes. For instance, memory can be improved by antagonizing GABA receptors (Table 1) or by antagonizing the NMDA receptor (Table 1); neurogenesis can be increased by drugs that interact with GSK3β, such as lithium (Table 1), or drugs that interact with the serotonergic system, such as fluoxetine (Tables 1, 2). The outcomes of the studies reported in Table 1 are summarized in Figure 3A. Importantly, 36 out of 58 interventions obtained the full rescue of the examined phenotype (Figure 3A, Rescued); 11 interventions obtained an improvement (Figure 3A, Improved); four interventions obtained the rescue of some of the examined phenotypes but not others (Figure 3A, Failed/Rescued); and only seven interventions were ineffective (Figure 3A, Failed). It must be observed that the studies reported in Table 1 used mice of different ages and treatments with different durations. Thus, it cannot be ruled out that the ineffectiveness of some treatments may be related to the age of mice and/or to an insufficient treatment duration. In addition, it must be emphasized that the results of treatment (“rescue,” “improvement” and “failure”) reported in the column “Outcome” of Table 1 refer to the specific phenotype indicated in the first column. We must be aware that the rescue of a given phenotypic feature may not necessarily lead to a cognitive improvement. Although we take these limitations into account, if we group together interventions that elicit a rescue or an improvement of the observed phenotype/s it ensures that 51 out of 58 interventions (88%) have a positive impact on the DS brain. We believe that this is an extremely important success that may give new hope for DS.

The question now arises as to how widely different approaches may produce the same result. It should be observed that the gene burden in DS alters numerous cellular pathways. Different signaling pathways concur, in many cases, to regulate the same cellular process. Thus, pharmacological restoration of a single pathway may be sufficient to correct a given defect. Consequently, therapies interacting with different pathways may ultimately lead to similar results. This aspect should not be disregarded, because the possibility to have a panel of effective therapies at hand will give us the opportunity to select the agent with as few side effects as possible.

Although animal models are essential for translation of drug findings from bench to bedside, we must be aware of possible limitations of the treatments attempted in mouse models in terms of translational value. The best validated animal model is not able to yield conclusive data when the experimental design is flawed or the execution of the study is not well-controlled. Yet, the studies reported in Tables 1, 2 were (a) conducted on the Ts65Dn mouse, which in spite of some limitations replicates many aspects of the human disease, and (b) targeted to molecular alterations or phenotypic features present in the model and in the DS brain. These studies may provide, therefore, a good starting point that, after better characterization of dosing, timing, and absence of short- and long-term side effects may help in the design of future clinical trials.

## Is there an optimum timing of therapies for DS?

**Figure 4:**
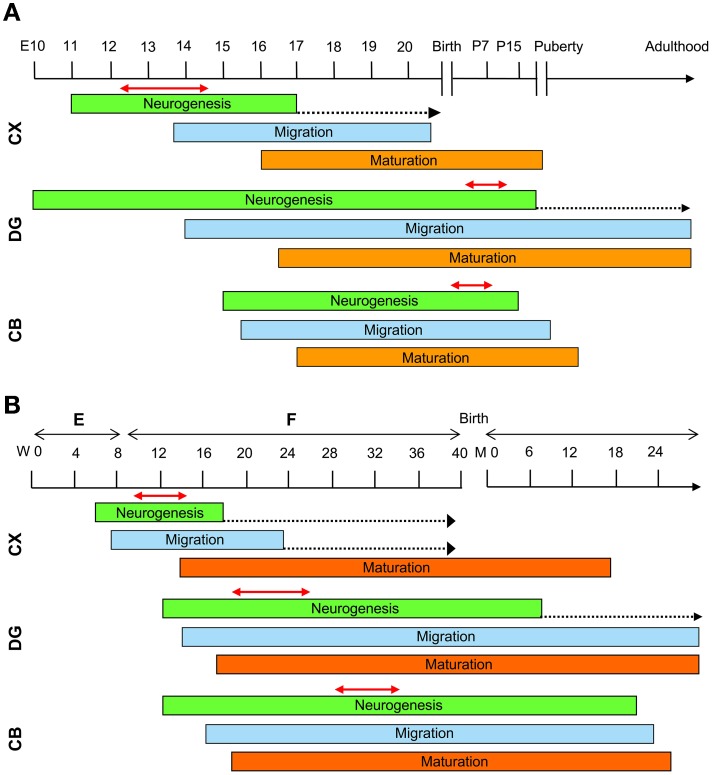
**Schematic representation of the timeline of brain development**. Timeline of mouse **(A)** and human **(B)** brain development. See text for explanations. The dotted arrows indicate a reduction in the rate of neurogenesis. The double-headed red arrows delineate the period of maximum neurogenesis in the different brain regions of the mouse and human brain. Abbreviations: CB, cerebellum; CX, neocortex; DG, dentate gyrys; E, embryonic; F, fetal; M, month; P, post-natal; W, week.

Most of the attempts to pharmacologically improve trisomy-linked brain alterations have been made in adult mice (compare Tables 1, 2). Figure 3B summarizes the number of studies in the Ts65Dn mouse models of DS that have tested the effects of pharmacotherapies at adult life stages, during the neonatal period, and during the embryonic period. Therapies were administered at adult life stages in 38 out of 55 studies (69%), in the neonatal period in eight studies (15%) and in the prenatal or prenatal + neonatal period in nine studies (16%). This striking imbalance deserves a comment. As hinted above, neurodevelopmental defects in people with DS (and mouse models of DS) are already present at fetal life stages. This is the period in which the bulk of neurogenesis takes place (Figure 4). There are two important exceptions to this rule: the hippocampal dentate gyrus and the cerebellum, two regions where granule neuron production largely occurs in the very early post-natal period. While in the hippocampal dentate gyrus neurogenesis goes on (at a slow rate) throughout life, in the cerebellum neurogenesis stops shortly after the early post-natal period (Figure 4). In view of the time course of brain development we can envisage that: (i) adult therapies may modulate ongoing hippocampal neurogenesis and, possibly, already existing hippocampal and extrahippocampal circuits. In addition, adult therapies may be used in order to prevent AD-linked neurodegeneration; (ii) neonatal therapies may largely shape hippocampal and cerebellar development; (iii) prenatal therapies may have by far the largest impact, by potentially affecting development of the whole brain (Figure 4). Therefore, we can expect that, while late therapies may modify the trisomic brain to a relatively limited extent, perinatal therapies are likely to exert more widespread effects, potentially affecting overall brain development. In the following sections we will summarize what we currently know about the efficacy of pharmacotherapies during different life stages in the Ts65Dn mouse model of DS. However, since this review intends to focus on the impact of early therapies, the effects of therapies at later life stages will only be briefly mentioned. For more details, the reader may refer to excellent recent reviews (Costa and Scott-McKean, 2013; Gardiner, 2015).

## Adult therapies

As mentioned above, most of the studies that have sought to pharmacologically improve the DS brain phenotype have mainly used adult mice. These studies focused on the hippocampus because hippocampal-dependent learning and memory are severely affected in DS. Table 1 summarizes the results of studies in adult mouse models of DS obtained during the past 14 years. Results of different therapies are grouped by the phenotypic features that have been examined. Since, in many instances, more than one feature has been taken into account in the same study, that study may appear more than once. The advantage of reporting results in this way is that i) the impact of different therapies on the same phenotypic feature and ii) the number of studies that have focused on that feature can be readily appreciated. Most of the studies on adult mice have examined the effect of treatment on learning and memory (L/M), without trying to find a mechanistic link between the behavioral effects and changes in the architecture and/or physiology of the hippocampal circuits. A few studies have examined, in addition to L/M, long-term potentiation (LTP) at hippocampal synapses, a form of synaptic plasticity that has been classically considered to be the electrophysiological correlate of learning and memory, although this view is becoming questionable (Abbas et al., 2015). Granule neurons of the hippocampal dentate gyrus continue to proliferate across life. The adult-produced granule neurons integrate into the hippocampal circuits and appear to play a role in memory performance (Imayoshi et al., 2008). However, relatively few studies have examined the effect of treatment on hippocampal neurogenesis. Signal processing depends on proper connectivity and thus, it is important to examine the effect of treatment on dendritic architecture and connectivity. However, there is a striking lack of information regarding this issue. A study in TgDyrk1A mice shows that EGCG restores, in addition to neurogenesis, granule cell dendritic architecture (Pons-Espinal et al., 2013) but, to our knowledge, only a single study has examined the effect of treatment on dendritic architecture in the Ts65Dn mouse (Table 1). The Ts65Dn mouse, similarly to individuals with DS, is bound to develop AD with age. Thus, it is of relevance to establish whether AD-like pathology can be pharmacologically improved. Accordingly, some studies have addressed this issue by specifically examining neurodegeneration (Table 1).

The lack of a common experimental protocol across the different research groups makes it difficult to compare the efficacy of different treatments. For instance, experiments vary for factors such as age of mice, doses, duration of treatment (acute/chronic) and experimental design. In addition, a limited number of trisomy-linked phenotypes were examined by most of these studies. Thus, the effect of treatment on the non-examined features remains to be established. Yet, by examining Table 1, it appears that 19 out of 36 interventions (53%) that examined L/M caused rescue of L/M, 7 (19%) caused an improvement and 10 (28%) had no effect; 10 out of 11 interventions that examined LTP caused rescue of LTP and one intervention caused an improvement; six out of eight interventions that examined neurogenesis caused restoration of neurogenesis. Thus, there is a large panel of treatments that is effective in rescuing/improving the major defects of the trisomic brain, at least in the Ts65Dn mouse model, although the clinical significance of acute treatments (see Table 1) remains to be established. A critical aspect that has been largely neglected is whether the effects of treatment outlast treatment cessation. Only six studies have taken this important issue into account and while two of them show that the effect of the selected therapy outlasts treatment cessation, the remaining four give disappointing results by showing that the effects disappear with time. The fact that the impact of a given therapy is ephemeral should not be disregarded, because continuous administration of drugs would be needed in order to maintain their effects, which might be impracticable.

### Future directions for adult therapies in DS

The studies summarized in Table 1 are promising in that they provide proof of principle demonstration that therapies can be attempted in adults with DS in order to improve learning and memory. Importantly, some of these studies have prompted clinical trials in individuals with DS (Table 3). Following the “pioneer” studies carried out so far, we believe that the issue of adult therapies in mouse models of DS should be readdressed in a more systematic manner in order to obtain pre-clinical results with a translational impact. (1) Druggable candidate molecules should be chosen. (2) Dosage and duration of treatment should be carefully established in order to avoid toxic effects. (3) Treatments should be administered at different times during adulthood, in order to establish whether their effect is age-dependent. (4) The effects of treatment should be examined at both the neuroanatomical and functional level, in order to establish the mechanism/s whereby a given therapy exerts its effects. (5) Evaluation of the effects of treatment should not be confined to the hippocampus but also extend to other brain regions, because changes in the synaptic organization of other brain structures may contribute to the beneficial effect of treatment. (6) Behavioral tests should be standardized. (7) The effects of a treatment should be examined after its discontinuation, in order to establish whether it leaves an enduring trace in the brain.

**Table 3:** **Clinical trials for intellectual disability in individuals with Down syndrome**.

**“A Study of RG1662 in Adults and Adolescents With Down Syndrome (CLEMATIS)”** (ClinicalTrials.gov Identifier: NCT02024789)
https://clinicaltrials.gov/ct2/show/NCT02024789
**“A Study of RG1662 in Individuals With Down Syndrome”** (ClinicalTrials.gov Identifier: NCT01436955)
https://ClinicalTrials.gov/show/NCT01436955
**“Down Syndrome Memantine Follow-up Study”** (ClinicalTrials.gov Identifier: NCT02304302)
https://clinicaltrials.gov/ct2/show/NCT02304302?cond=%22Down+Syndrome%22&rank=40
**“Efficacy and Safety of Memantine Hydrochloride in Enhancing the Cognitive Abilities of Young Adults With Down Syndrome”** (ClinicalTrials.gov Identifier: NCT01112683)
https://ClinicalTrials.gov/show/NCT01112683
**“Memantine and Down's Syndrome”** (ClinicalTrials.gov Identifier: NCT00240760)
https://ClinicalTrials.gov/show/NCT00240760
**“Down Syndrome Memantine Follow-up Study”** (ClinicalTrials.gov Identifier: NCT02304302)
https://ClinicalTrials.gov/show/NCT02304302
**“Evaluating The Safety Of Donepezil Hydrochloride (Aricept) For Up To 1 Year In The Treatment Of The Cognitive Dysfunction Exhibited By Children With Down Syndrome—Follow-Up To A 10-Week, Double-Blind, Placebo-Controlled Trial”** (ClinicalTrials.gov Identifier:NCT00675025). https://clinicaltrials.gov/ct2/show/record/NCT00675025?term=%22down+syndrome%22+AND+%22clinical+trial%22&rank=4
**“Evaluating The Efficacy And Safety Of Donepezil Hydrochloride (Aricept) In The Treatment Of The Cognitive Dysfunction Exhibited By Children With Down Syndrome, Aged 6 To 10”** (ClinicalTrials.gov Identifier: NCT00754013)
https://ClinicalTrials.gov/show/NCT00754013
**“Evaluating The Efficacy And Safety Of Donepezil Hydrochloride (Aricept) In Treating Cognitive Dysfunction Exhibited By Children With Down Syndrome”** (ClinicalTrials.gov Identifier: NCT00570128)
https://ClinicalTrials.gov/show/NCT00570128
**“Rivastigmine Study in Adolescents With Down Syndrome (DS-Riv)”** (ClinicalTrials.gov Identifier: NCT01084135)
https://clinicaltrials.gov/ct2/show/NCT01084135?term=down+syndrome&rank=35
**“Efficacy of Rivastigmine in Patients With Down Syndrome”** (ClinicalTrials.gov Identifier: NCT00748007)
https://ClinicalTrials.gov/show/NCT00748007
**“Egcg, a dyrk1a Inhibitor as Therapeutic Tool for Reversing Cognitive Deficits in Down Syndrome Individuals”** (ClinicalTrials.gov Identifier: NCT01394796)
https://clinicaltrials.gov/ct2/show/NCT01394796
**“Normalization of dyrk1A and APP Function as an Approach to Improve Cognitive Performance and Decelerate AD Progression in DS Subjects: Epigallocatechin Gallate as Therapeutic Tool”** (ClinicalTrials.gov Identifier: NCT01699711)
https://ClinicalTrials.gov/show/NCT01699711
**“Vitamin E in Aging Persons With Down Syndrome”** (ClinicalTrials.gov Identifier: NCT00056329)
https://clinicaltrials.gov/ct2/show/NCT00056329
**“Multicenter Vitamin E Trial in Aging Persons With Down Syndrome”** (ClinicalTrials.gov Identifier: NCT01594346)
https://ClinicalTrials.gov/show/NCT01594346

*The clinical trials reported investigate the efficacy of RG1662 (a GABA_A_α5 negative allosteric modulator), memantine (antagonist of the NMDA receptor), Donepezil (AChE inhibitor), Rivastigmine (AChE inhibitor), EGCG (Inhibitor of DYRK1A kinase), and Vitamin E (Antioxidant) on cognitive performance in children or adults with Down syndrome*.

## Early therapies

Investigations into early therapies for DS are much less abundant in comparison with the numerous studies regarding adult therapies (see Figure 3B). However, the few available studies show that perinatal therapies have impressive effects on the trisomic brain and that they can rescue numerous trisomy-linked brain alterations such as neurogenesis, brain cellularity, dendritic development, connectivity, and behavior.

### Neonatal therapies

Table 2 shows that the three treatments that have been used so far in neonate Ts65Dn mice (SAG, fluoxetine, and EGCG) have a positive impact on development of the cerebellum (SAG) and hippocampus (fluoxetine and EGCG).

#### SAG

The cerebellum is disproportionately small in the Ts65Dn mouse and in individuals with DS and has a reduced number of granule neurons and Purkinje cells. A first pioneer study examined the effect of SAG, a synthetic activator of the Sonic hedgehog (Shh) pathway, on cerebellar neurogenesis in newborn mice (Roper et al., 2006). In rodents, most cerebellar granule neurons are produced within the first two post-natal weeks, with a peak within the first few post-natal days (Sillitoe and Joyner, 2007; Sudarov and Joyner, 2007). Trisomic granule cell precursors show a reduced response to the Sonic hedgehog protein signal *in vitro* (Roper et al., 2006), demonstrating that this is a cell-autonomous deficit. In trisomic mice a single systemic treatment with SAG at birth was found to increase neurogenesis and restore granule cell precursor populations when mice were tested at 6 days old (Roper et al., 2006). These are the first results demonstrating that an early therapy can fully reinstate defective generation of cerebellar granule neurons. A subsequent study showed that the effect of a single neonatal injection of SAG resulted in normal cerebellar morphology in tests carried out when mice reached 4 months of age (Das et al., 2013). In contrast, 6 days after a single neonatal injection of SAG, there was no improvement in the dentate gyrus proliferation deficit in Ts65Dn mice (Das et al., 2013), suggesting that SAG may differentially affect different neural precursor cell populations. Yet, neonatal treatment with SAG restored performance in a hippocampal-dependent task (MWM) and LTP at the synapse Schaffer collaterals-CA1 when mice were 4 months old (Das et al., 2013). This evidence suggests that Shh has a role, that remains to be defined, in perinatal hippocampal development, and indicates a long-lasting effect of treatment on hippocampal function, apparently independently from neurogenesis normalization. In a more recent study, newborn mice received a single injection of SAG and were examined at 4 months of age for cerebellum-dependent learning (Gutierrez-Castellanos et al., 2013). Despite the positive impact of SAG on cerebellar neuroanatomical architecture, SAG treatment failed to rescue long-term cerebellar-based learning in mice aged 4 months. The lack of effect may be attributable to the persistence of altered granule cell electrophysiological properties and to the fact that in Ts65Dn mice there are fewer Purkinje cells, the proliferation of which cannot be affected by treatment in view of their embryonic birth date (Sillitoe and Joyner, 2007; Sudarov and Joyner, 2007).

#### Fluoxetine

The hippocampus of Ts65Dn mice and individuals with DS is reduced in size due to severe neurogenesis alterations and dendritic hypotrophy. The serotonergic system, which is altered in DS, plays a fundamental role in neurogenesis and dendritic development and, similarly to humans with DS, Ts65Dn mice exhibit reduced expression of the serotonin 5-HT1A receptor. Therefore, we wondered whether neonatal treatment with fluoxetine, a selective serotonin reuptake inhibitor, was able to rescue hippocampal neurodevelopmental alterations. We found that, immediately after a brief neonatal treatment (from P3 to P15) with fluoxetine, hippocampal neurogenesis, and total granule cell number were fully normalized (Bianchi et al., 2010b). Importantly, 1 month after treatment cessation, treated Ts65Dn mice exhibited fully restored granule cell number, restoration of granule cell dendritic pattern, hippocampal connectivity, signal transfer from the granule cells to CA3, and hippocampal-dependent memory function (Bianchi et al., 2010b; Guidi et al., 2013; Stagni et al., 2013). In a subsequent study we examined the effects of neonatal treatment with fluoxetine when mice reached adulthood (3 months of age) and found that in neonatally-treated Ts65Dn mice hippocampal cellularity, dendritic architecture, spine density, and memory functions were still fully rescued (Stagni et al., 2015). Moreover, we found that the increased levels of the APP-derived βCTF peptide in adult Ts65Dn mice were normalized following neonatal treatment with fluoxetine. This effect was accompanied by restoration of endosomal abnormalities, a βCTF-dependent feature of DS and AD. These results show that not only does early treatment with fluoxetine enduringly restore cognitive impairment but it may also prevent early signs of AD-like pathology.

#### EGCG

Among HSA21 genes known to influence brain development, *Dyrk1A* is one of the potent candidate genes closely implicated in the DS neurological phenotype. Transgenic mice that overexpress Dyrk1A exhibit brain developmental defects and behavioral alterations similar to those found in DS patients and in murine models with partial MMU16 trisomies, such as the Ts65Dn mouse, which carries extra copies of several genes, including the *Dyrk1A* gene (De la Torre et al., 2014). These observations suggest that therapeutic strategies, aimed to modulate DYRK1A activity may also have a positive effect in DS. EGCG is one of the most specific inhibitors of DYRK1A kinase activity. We are currently examining the effect of epigallocatechin-3-gallate (EGCG), the major catechin in green tea on hippocampal development. This phytochemical may have fewer side effects in comparison with SAG or fluoxetine. Our results show that neonatal treatment with EGCG fully restores hippocampal neurogenesis and cellularity (Stagni et al., 2014). The duration of these effects still remains to be elucidated.

### Prenatal therapies

Five different types of prenatal therapies have been used so far in DS mouse models, four of which have a positive effect on numerous neurodevelopmental alterations (Table 2).

#### Choline

Cholinergic neurons provide the primary source of acetylcholine, a fundamental brain neurotransmitter. A common trait of DS and AD individuals and the Ts65Dn mouse model is the degeneration of the Basal Forebrain Cholinergic Neurons (BFCNs). This group of neurons is important for (i) explicit memory function, subserved by projections from the medial septal nucleus to the hippocampus and (ii) attention and working memory, subserved by projections from the nucleus basalis to the frontal cortex. In Ts65Dn mice degeneration of the BFCNs begins at 6 months of age, and, similarly to humans with DS and AD, continues during adulthood. Based on the unavoidable degeneration of BFCNs in these pathologies, a series of related studies (Moon et al., 2010; Velazquez et al., 2013; Ash et al., 2014; Kelley et al., 2014) considered the hypothesis that improvement of BFCNs may prevent the defects related to their degeneration. Moon et al. (2010) supplemented the diet of pregnant Ts65Dn females with high concentrations (> 4.5-fold than normal) of choline, beginning at E1 and continuing during lactation until the pups were weaned at P21. This regimen had previously been shown to have several benefits on normal rodents: (i) organizational improvement on BFC neuronal systems, ii) enduring enhancement of cognitive functions (i.e., explicit memory and attention), and iii) neuroprotection against neural insults (see Moon et al., 2010). The effect of treatment on the progeny of Ts65Dn mothers supplemented with choline was evaluated starting from when mice were 6 months of age. Behavioral testing was then continued for the following 6 months (Moon et al., 2010). In order to establish whether treatment improved cognitive performance, mice were tested with a five-choice visual discrimination task. Results showed that increasing maternal choline intake during pregnancy and lactation significantly ameliorates attentional functioning of the trisomic offspring, albeit not completely. In a subsequent work (Velazquez et al., 2013) the same schedule of treatment as in Moon et al.'s study was used, plus environmental enrichment, and mice were examined when they were 13–17 months of age. Choline supplementation was found to restore hippocampal neurogenesis (evaluated with doublecortin immunostaining) and hippocampal-dependent spatial cognition, tested with the Radial Arm Water Maze. Two subsequent studies examined the effect of the same treatment on the BFCNs in mice aged 4.3–7.5 and 13–17 months (Ash et al., 2014; Kelley et al., 2014). A reduction in the number of BFCNs was found in the medial septum of Ts65Dn mice aged 13–17 months. This defect was improved by treatment (Ash et al., 2014). These findings indicate that embryonic/early post-natal choline supplementation has effects that extend to very advanced life stages. Although the mechanisms by which prenatal/neonatal supplementation of choline reinstates hippocampal neurogenesis and functions in the Ts65Dn mouse remain to be elucidated, some theories were formulated by Moon et al. (2010) and Velazquez et al. (2013). It is possible that choline mediates these beneficial effects, altering the DNA methylation status (epigenetic effects) or regulating the production of phospholipid components of membranes. Although these theories are suggestive, we know too little about the molecular mechanism of choline in DS and further studies are needed to solve these questions.

#### Fluoxetine

Since serotonin is essential for neurogenesis and dendritic development (Faber and Haring, 1999; Whitaker-Azmitia, 2001), we hypothesized that treatment with fluoxetine during pregnancy could rescue most of the neurodevelopmental alterations that characterize the trisomic brain. We treated pregnant Ts65Dn females from E10 to delivery with the aim of restoring the bulk of neurogenesis. We found that untreated Ts65Dn pups exhibited a severe neurogenesis reduction and hypocellularity throughout the forebrain (subventricular zone, subgranular zone, neocortex, striatum, thalamus, hypothalamus), midbrain (mesencephalon) and hindbrain (cerebellum and pons). In Ts65Dn mice embryonically-treated with fluoxetine precursor proliferation and cellularity were fully restored in all these regions. Furthermore, embryonic treatment with fluoxetine restored the expression of the 5-HT1A receptor in the subventricular zone and hippocampal regions of Ts65Dn mice (Guidi et al., 2014). To verify whether prenatal treatment with fluoxetine had enduring effects, we examined the offspring of treated and untreated mothers when mice reached 45 days of age, i.e., at 1.5 months after treatment cessation. We found that neural precursor proliferation was still restored in the two major post-natal brain neurogenic niches (subventricular zone and subgranular zone of the dentate gyrus) (Guidi et al., 2014). In addition, in the hippocampal dentate gyrus the typical reduction in neurogenesis and the relative increase in astrogliogenesis were fully corrected indicating a long-term effect on the differentiation program. The total number of granule neurons was also still restored. Furthermore, in embryonically-treated Ts65Dn mice the dendritic development of post-natally born granule neurons was normalized with full correction of the severe dendritic hypotrophy that characterizes the trisomic condition. The counterpart of this effect was restoration of pre- and post-synaptic terminals. Finally, embryonically- treated Ts65Dn mice aged 45 days exhibited restoration of cognitive performance, indicating that the positive impact of embryonic treatment on brain development was functionally effective in adulthood.

#### NAP+SAL

Activity-dependent neuroprotective protein (ADNP) and activity-dependent neurotrophic factor (ADNF) are essential for brain formation (Incerti et al., 2011). The active peptide fragments of these proteins, NAPVSIPQ (NAP) and SALLRSIPA (SAL), mimic the activity of their parent proteins. These peptides have been shown to exert a protective effect against oxidative stress, the severity of traumatic head injury, stroke, and toxicity associated with the Aβ peptide, and to stabilize and repair microtubules (Gozes et al., 2005, 2008). A preliminary study showed that prenatal treatment (in the period E8–E12) with NAP+SAL prevents the delay of neurodevelopmental milestones in trisomic offspring (Toso et al., 2008). At a cellular level, prenatal NAP+SAL restore altered subunits of the NMDA receptor and GABA_A_ receptor (Vink et al., 2009), suggesting that one mechanism by which treatment exerts its effect may be the normalization of the efficacy of excitatory and inhibitory pathways. In a subsequent study the effect of prenatal treatment (in the period E8–E12) with NAP+SAL on learning and memory was examined when the offspring had reached 8–10 months of age (Incerti et al., 2012). Prenatally-treated Ts65Dn mice exhibited a learning curve that was similar to that of untreated euploid mice. Unfortunately, the results of the probe test are not mentioned and thus it is not possible to establish the effect of this treatment on memory. Moreover, the study did not examine the effects of treatment on neurogenesis and overall brain development. However, the results prospect the possibility of potential pregnancy interventions for DS with these peptides.

#### SGS-111

Neurons of DS patients exhibit a three- to four-fold increase in intracellular reactive oxygen species (ROS) due to over expression of SOD1, the gene that is responsible for the formation of the enzyme superoxide dismutase that changes oxygen free radicals into hydrogen peroxide. This oxidative stress, which damages mithocondrial membrane and lipids, occurs in DS during pre- and post-natal development and can modify critical processes of neurogenesis, differentiation, migration, and survival. Therefore, oxidative stress has been linked to the brain abnormalities observed in DS. Since oxidative stress has been reported as early as in the fetal stage, SGS-111, an analog of piracetam with neuroprotective and nootropic properties, was administered to pregnant Ts65Dn females from the day of conception, throughout pregnancy, and to their pups during the following 5 months (Rueda et al., 2008b). The behavioral characterization carried out at the end of treatment showed that chronic administration of the antioxidant SGS-111 reduced the hyperactivity shown by Ts65Dn mice but failed to improve learning and memory. The lack of effects may be due to the fact that in Ts65Dn mice the MWM task is relatively independent of the neurotoxic effect of increased oxidative stress.

#### Tocopherol

Another important aspect of oxidative stress found in DS brains is lipid damage caused by elevated levels of lipid peroxidation. It has been reported that the concentration of isoprostanes (a marker for lipid peroxidation) in the amniotic fluid of mothers who were pregnant with DS fetuses was nine times greater than in pregnancies involving normal fetuses, suggesting that lipid peroxidation occurs early in pregnancy (Perrone et al., 2007). Therefore, the antioxidant α-tocopherol, the most biologically active form of vitamin E, was chronically administered to pregnant Ts65Dn females from the day of conception throughout the pregnancy and to their pups until adulthood, in order to prevent the developmental consequences of elevated oxidative stress (Shichiri et al., 2011). Supplementation of α-tocopherol was found to reduce acroleine, a lipid peroxidation product, in the dentate gyrus of adult Ts65Dn mice and this effect was accompanied by an increase in granule cell density. In addition, treatment ameliorated abnormal anxiety/regardlessness in the Elevated-Plus Maze task in Ts65Dn mice, improved spatial learning, and partially improved retention memory in the MWM test. No effect of treatment on hyperactivity was found in the spontaneous motor activity test.

#### EGCG

Although this review is focused on therapies in the Ts65Dn mouse model, we will briefly report data obtained in the transgenic YACtg152F7 mouse, a strain that over expresses DYRK1A kinase, in view of the potential impact for DS. Transgenic YACtg152F7 mice were treated with two different polyphenol-based diets, from gestation to adulthood (Guedj et al., 2009). Chronic administration of polyphenols from green tea (that include EGCG) was found to correct, in adult transgenic mice, brain weight, and thalamus-hypothalamus volume alterations that are strongly related to *Dyrk1a* gene copy number. Moreover, this treatment restored hippocampal mRNA levels for the neurotrophic factor BDNF and its plasma membrane receptor TrkB. Consistently with the positive effect of treatment on these markers of synaptic plasticity, long-term memory, assessed using the Novel Object Recognition test, was completely restored in treated transgenic mice.

## Timing is all

The studies carried out in mouse models at adult life stages show that it is possible to improve or even rescue hippocampal-dependent learning and memory, although the duration of these effects still remains a matter of investigation in the majority of cases. After the period of neuron proliferation and maturation, which takes place in the prenatal and neonatal period, there is no means to increase the number of neurons forming the brain, except—to a limited extent—for the hippocampal dentate gyrus. Thus, after the critical periods of neurogenesis and synaptogenesis the brain can undergo relatively limited plastic changes and late therapies are unlikely to exert drastic changes in the brain. Yet, although late therapies may exert a limited benefit, even a partial improvement of ID in adults with DS and/or prevention of AD development would be an extremely important achievement. Importantly, the results reviewed above clearly show that therapies administered during the early stages of brain development have an extremely pronounced effect on the trisomic brain in terms of the phenotypic features that they are able to rescue and in terms of the duration of their effects. The studies in DS mouse models provide proof of principle evidence that it might be possible to rescue brain development provided that treatments are administered during the earliest phases of brain development. The magnitude and striking persistence of the effects of neonatal and prenatal interventions emphasizes the importance of early treatment in DS.

The normal ontogeny of neural development in rodents is different from humans because rodents have considerable post-natal development and humans have considerably more prenatal maturation of their nervous systems (Figure 4). This aspect is fundamental to the planning of a correct pharmacological intervention during a specific phase of brain development. In mice, cortical neurogenesis takes place between embryonic days E11–E17 (Takahashi et al., 1996) (Figure 4A). At birth, except for a few specialized regions, including the subventricular zone/rostral migratory stream, the hippocampal dentate gyrus and the cerebellar cortex, the brain enters a state of replicative quiescence. In the hippocampal dentate gyrus, although neurogenesis begins at E10 it exhibits its maximum rate in the first two post-natal weeks and then continues at a slow rate throughout life (Altman and Bayer, 1975, 1990a,b) (Figure 4A). In the mouse cerebellum, granule cell production begins at approximately E15 and is accomplished by the second post-natal week (Sillitoe and Joyner, 2007; Sudarov and Joyner, 2007) (Figure 4A). In the human brain, after the formation of the neural tube, (by gestational week 3), neural progenitors produce neurons that migrate from the ventricular zone, the primitive epithelial sheet of dividing neural progenitor cells, to their final destination in the regions that will form the different brain parts. In the human forebrain neocortical neurons are generated during a restricted period that begins at approximately gestational week 6 and is largely completed by week 18 (Stiles and Jernigan, 2010) (Figure 4B). After their final division, postmitotic neurons migrate outward from the VZ and once they have reached their target regions develop axons and dendrites and begin to form synaptic connections. Synaptic production continues during the first two post-natal years (Figure 4B). In the human dentate gyrus, neurogenesis begins at approximately gestational week 12 and is almost accomplished within the first post-natal year (Seress et al., 2001; Rice and Barone, 2010), although, similarly to rodents, it continues at a slow rate throughout life (Eriksson et al., 1998) (Figure 4B). Production of cerebellar granule cells starts at gestational week 12 (ten Donkelaar et al., 2003) and continues in the first few post-natal months (Abráham et al., 2001) (Figure 4B). Noninvasive prenatal testing (NIPT) for DS, using massively parallel sequencing of maternal plasma DNA, facilitates early detection of affected fetuses. As envisaged by Guedj et al., if NIPT is performed at approximately 12 weeks of pregnancy there is a potential 28-week window of opportunity in which to treat the fetus by orally administering small molecules to the mother (Guedj and Bianchi, 2013; Guedj et al., 2014). Considering the timeline of brain development, treatment during weeks 12–16 of pregnancy may have a large impact on cortical neurogenesis (Figure 4B). Treatments after week 16 may principally modulate cortical neuron maturation and synapse formation. Finally, treatment during late pregnancy and the first years of life may have a large impact on neurogenesis in the hippocampal dentate gyrus and cerebellum. Demonstration, obtained in mouse models, that the defects of the DS brain are reversible opens a breakthrough for the prevention of intellectual disability. The timeline of human brain development (Figure 4B) shows that there are windows of opportunity that can be exploited in order to pharmacologically improve (and hopefully, rescue) the neurodevelopmental alterations that characterize the DS brain.

## Translational impact of studies in mouse models

The discovery that early pharmacotherapies can restore brain development in mouse models of DS raises the question of the translation of these results to human beings with DS. When designing prenatal or neonatal treatments for DS two important issues must be taken into account: the placental (and blood-brain) barrier and the possible toxicity of treatment. The drugs used so far in mice cross the placental and brain barrier but their use may pose some caveats in view of potential side effects. Pharmacological stimulation of the Shh pathway with SAG in newborn infants as a therapeutic strategy might be problematic. Since chronic Shh pathway stimulation is observed in a number of tumor types, a better understanding of the side effects of Shh treatment is required. Fluoxetine, which is an antidepressant prescribed in adults and adolescents, may be safer than SAG. Although it is in clinical trial in children as a treatment for various behavioral disturbances (Alcamí Pertejo et al., 2000; DeLong et al., 2002; Hollander et al., 2005), possible side effects in neonates cannot be ruled out. Fluoxetine use in early pregnancy has been associated with a slightly increased risk of specific cardiovascular malformations (Reefhuis et al., 2015). However, another recent study conducted on a large cohort of subjects (approximately 36,700 exposed infants and 2,200,000 unexposed infants) indicates that there is not a substantial teratogenic effect of SSRI, including fluoxetine, during the first trimester of pregnancy (Furu et al., 2015). Exposure to antidepressants (including fluoxetine) during the second and third trimester does not have substantial effects on milestones of development (Einarson et al., 2009; Pedersen et al., 2010). However, the potential risk of pre-term birth (Hayes et al., 2012) and pulmonary hypertension in the neonate (Chambers et al., 2006; Olivier et al., 2013) cannot be ruled out. It must also be observed that *in utero* exposure to serotonin reuptake inhibitors may result in a neonatal withdrawal syndrome (Moses-Kolko et al., 2005; Sanz et al., 2005). Though the withdrawal effect is generally self-limited, this aspect must be taken into account. Considering the impressive effects of fluoxetine in a mouse model of DS, the side effects of prenatal exposure to fluoxetine may be considered a relatively minor problem in the face of the possible rescue of cognitive disability. At present, there are no published data on DS babies born from mothers taking fluoxetine (or other antidepressants). A pilot feasibility trial of perinatal fluoxetine treatment at the Southwestern Medical Center of the University of Texas was approved in 2014 and its start is scheduled for 2015 (Byerly, M., Carlin, M. and Horsager-Boehrer, R., 2014. A Pilot Feasibility Trial of Prenatal and Early Post-natal Fluoxetine Treatment for Intellectual Impairments of Down Syndrome https://wwwutswmedicineorg/stories/articles/year-2015/down-syndromehtml). EGCG is a phytochemical derived from green tea extracts. The use and dosage of substances that derive from plants as natural remedies for various diseases is deeply rooted in the history of mankind. Therefore, natural substances may represent attractive tools for the therapy of various disturbances, including DS. EGCG appears to be a safe phytochemical (Vacca and Valenti, 2015) and its use has numerous beneficial health effects (Kim et al., 2014). EGCG is often classified as an antioxidant but it may function as a pro-oxidant in some cellular contexts. EGCG has many actions that do not depend on anti-oxidant mechanisms, including direct interaction with proteins and phospholipids in the plasma membrane, and regulation of signal transduction pathways and transcription factors (Kim et al., 2014). It has been shown that high doses of EGCG have hepatotoxic effects (Lambert et al., 2009). However, the doses used in pre-clinical studies in mouse models (De la Torre et al., 2014; Stagni et al., 2014) and in the clinical trials with EGCG (reported in Table 3) are well below those that are known to cause adverse effects. EGCG administered to pregnant rats does not have teratogenic effects (Isbrucker et al., 2006). It is not known whether EGCG may have adverse effects during pregnancy in humans. A clinical trial for young adults with DS (De la Torre et al., 2014) shows that the positive effect of treatment with EGCG on behavior tends to disappear with time. We found that neonatal treatment with EGCG rescued hippocampal development in the Ts65Dn mouse model, similarly to that with fluoxetine. At this point it is of paramount importance to establish whether EGCG administered prenatally can rescue overall brain development, similarly to fluoxetine, and whether this effect is retained with time. If so, EGCG may be a promising treatment for the prevention of ID in DS. The neuroprotective peptides NAP and SAL can be orally administered. Moreover, NAP penetrates cells and crosses the blood-brain barrier after nasal or systemic administration. This would make treatment of individuals with DS easily feasible. These peptides do not seem to have adverse effects in animal models, and functional behavioral assays in rats show no adverse side effects with NAP concentrations that are approximately 500-fold higher than the biologically active dose (see Gozes et al., 2008). The beneficial effects of embryonic treatment on learning and memory in the Ts65Dn mouse model suggest that these peptides may be employed for prenatal treatment in DS. Choline and vitamin E are important supplements that should be taken in adequate amounts, and choline in large amounts appears to be required during pregnancy to support fetal development (Yan et al., 2013). No toxic or teratogenic effects are reported in the literature following an intake of the recommended daily range dosage of choline and vitamin E. Thus, choline and vitamin E are not likely to cause adverse effects on fetuses or babies with DS. Embryonic treatment with vitamin E improves spatial learning and delays the onset of cognitive and morphological brain abnormalities in the Ts65Dn mouse model (Shichiri et al., 2011). Although vitamin E may represent a safe and effective treatment during pregnancy, its actions appear less prominent in comparison with those of other agents. Therefore, it may be useful to combine other treatments with vitamin E in order to obtain a more significant outcome. Embryonic/early post-natal choline supplementation was found to restore behavior when mice were aged 13–17 months (Velazquez et al., 2013). Since choline is considered to be a very safe nutrient, it may be used for prenatal treatment for DS. However, further studies are needed in order to establish whether choline restores the neurodevelopmental defects of the DS brain in addition to preventing age-related cognitive deterioration. No data are available regarding potential toxic effects of SGS-111 during pregnancy, and the effects of early treatment with SGS-111 are less prominent in comparison with those of other agents.

## Conclusion

The exciting discovery that the brain abnormalities of mouse models of DS can be prevented with early interventions gives us reason to believe that treatments during pregnancy may rescue brain development in fetuses with DS. Importantly, three reported cases of DS babies whose mothers took high doses of vitamin B (plus other substances) during pregnancy provide encouraging results (Baggot and Baggot, 2014) and strengthen the idea that early therapies for DS may have a very positive impact on ID. For this reason we deem it extremely important to expedite the discovery of additional therapies practicable in humans, in order to identify the best treatment/s in terms of efficacy and paucity of side effects. Prompt achievement of this goal is the big challenge for the scientific community of researchers interested in DS.

### Conflict of interest statement

The authors declare that the research was conducted in the absence of any commercial or financial relationships that could be construed as a potential conflict of interest.

## References

[B1] Abbas A. K. Villers A. Ris L. (2015). Temporal phases of long-term potentiation (LTP): myth or fact? Rev. Neurosci.. 26, 507–546. 10.1515/revneuro-2014-0072 25992512

[B2] Abráham H. Tornóczky T. Kosztolányi G. Seress L. (2001). Cell formation in the cortical layers of the developing human cerebellum. Int. J. Dev. Neurosci. 19, 53–62. 10.1016/S0736-5748(00)00065-4 11226755

[B3] Ahmed M. M. Dhanasekaran A. R. Block A. Tong S. Costa A. C. Stasko M.. (2015). Protein dynamics associated with failed and rescued learning in the Ts65Dn mouse model of Down syndrome. PLoS ONE 10:e0119491. 10.1371/journal.pone.0119491 25793384 PMC4368539

[B4] Alcamí Pertejo M. Peral Guerra M. Gilaberte I. (2000). Open study of fluoxetine in children with autism. Actas Esp. Psiquiatr. 28, 353–356. 11262279

[B5] Altman J. Bayer S. (1975). Postnatal development of the hippocampal dentate gyrus under normal and experimental conditions, in The Hippocampus, Vol. 1, eds Isaacson R. L. Pribram K. H. (New York, NY; London: Plenum Press), 95–122.

[B6] Altman J. Bayer S. A. (1990a). Mosaic organization of the hippocampal neuroepithelium and the multiple germinal sources of dentate granule cells. J. Comp. Neurol. 301, 325–342. 10.1002/cne.903010302 2262594

[B7] Altman J. Bayer S. A. (1990b). Migration and distribution of two populations of hippocampal granule cell precursors during the perinatal and postnatal periods. J. Comp. Neurol. 301, 365–381. 10.1002/cne.903010304 2262596

[B8] Ash J. A. Velazquez R. Kelley C. M. Powers B. E. Ginsberg S. D. Mufson E. J.. (2014). Maternal choline supplementation improves spatial mapping and increases basal forebrain cholinergic neuron number and size in aged Ts65Dn mice. Neurobiol. Dis. 70, 32–42. 10.1016/j.nbd.2014.06.001 24932939 PMC4133151

[B9] Baggot P. J. Baggot R. M. (2014). Fetal therapy for Down syndrome: report of three cases and a review of the literature. J. Am. Phys. Surg. 19, 20–24. 29108162

[B10] Bartesaghi R. Guidi S. Ciani E. (2011). Is it possible to improve neurodevelopmental abnormalities in Down syndrome? Rev. Neurosci. 22, 419–455. 10.1515/rns.2011.037 21819263

[B11] Becker L. Mito T. Takashima S. Onodera K. (1991). Growth and development of the brain in Down syndrome. Prog. Clin. Biol. Res. 373, 133–152. 1838182

[B12] Begenisic T. Baroncelli L. Sansevero G. Milanese M. Bonifacino T. Bonanno G.. (2014). Fluoxetine in adulthood normalizes GABA release and rescues hippocampal synaptic plasticity and spatial memory in a mouse model of Down syndrome. Neurobiol. Dis. 63, 12–19. 10.1016/j.nbd.2013.11.010 24269730

[B13] Belichenko P. V. Masliah E. Kleschevnikov A. M. Villar A. J. Epstein C. J. Salehi A.. (2004). Synaptic structural abnormalities in the Ts65Dn mouse model of Down Syndrome. J. Comp. Neurol. 480, 281–298. 10.1002/cne.20337 15515178

[B14] Benavides-Piccione R. Ballesteros-Yáñez I. de Lagrán M. M. Elston G. Estivill X. Fillat C.. (2004). On dendrites in Down syndrome and DS murine models: a spiny way to learn. Prog Neurobiol. 74, 111–126. 10.1016/j.pneurobio.2004.08.001 15518956

[B15] Bianchi P. Ciani E. Contestabile A. Guidi S. Bartesaghi R. (2010a). Lithium restores neurogenesis in the subventricular zone of the Ts65Dn mouse, a model for down syndrome. Brain Pathol. 20, 106–118. 10.1111/j.1750-3639.2008.00246.x 19298631 PMC8094672

[B16] Bianchi P. Ciani E. Guidi S. Trazzi S. Felice D. Grossi G.. (2010b). Early pharmacotherapy restores neurogenesis and cognitive performance in the Ts65Dn mouse model for Down syndrome. J. Neurosci. 30, 8769–8779. 10.1523/JNEUROSCI.0534-10.2010 20592198 PMC6632890

[B17] Blanchard J. Bolognin S. Chohan M. O. Rabe A. Iqbal K. Grundke-Iqbal I. (2011). Rescue of synaptic failure and alleviation of learning and memory impairments in a trisomic mouse model of down syndrome. J. Neuropathol. Exp. Neurol. 70, 1070–1079. 10.1097/NEN.0b013e318236e9ad 22082658

[B18] Chakrabarti L. Best T. K. Cramer N. P. Carney R. S. Isaac J. T. Galdzicki Z.. (2010). Olig1 and Olig2 triplication causes developmental brain defects in Down syndrome. Nat. Neurosci. 13, 927–934. 10.1038/nn.2600 20639873 PMC3249618

[B19] Chakrabarti L. Galdzicki Z. Haydar T. F. (2007). Defects in embryonic neurogenesis and initial synapse formation in the forebrain of the Ts65Dn mouse model of Down syndrome. J. Neurosci. 27, 11483–11495. 10.1523/JNEUROSCI.3406-07.2007 17959791 PMC6673208

[B20] Chambers C. D. Hernandez-Diaz S. Van Marter L. J. Werler M. M. Louik C. Jones K. L.. (2006). Selective serotonin-reuptake inhibitors and risk of persistent pulmonary hypertension of the newborn. N. Engl. J. Med. 354, 579–587. 10.1056/NEJMoa052744 16467545

[B21] Chang Q. Gold P. E. (2008). Age-related changes in memory and in acetylcholine functions in the hippocampus in the Ts65Dn mouse, a model of Down syndrome. Neurobiol. Learn. Mem. 89, 167–177. 10.1016/j.nlm.2007.05.007 17644430 PMC2246382

[B22] Clark S. Schwalbe J. Stasko M. R. Yarowsky P. J. Costa A. C. (2006). Fluoxetine rescues deficient neurogenesis in hippocampus of the Ts65Dn mouse model for Down syndrome. Exp. Neurol. 200, 256–261. 10.1016/j.expneurol.2006.02.005 16624293

[B23] Colas D. Chuluun B. Warrier D. Blank M. Wetmore D. Z. Buckmaster P.. (2013). Short-term treatment with the GABAA receptor antagonist pentylenetetrazole produces a sustained pro-cognitive benefit in a mouse model of Down's syndrome. Br. J. Pharmacol. 169, 963–973. 10.1111/bph.12169 23489250 PMC3696321

[B24] Contestabile A. Fila T. Ceccarelli C. Bonasoni P. Bonapace L. Santini D.. (2007). Cell cycle alteration and decreased cell proliferation in the hippocampal dentate gyrus and in the neocortical germinal matrix of fetuses with Down syndrome and in Ts65Dn mice. Hippocampus 17, 665–678. 10.1002/hipo.20308 17546680

[B25] Contestabile A. Greco B. Ghezzi D. Tucci V. Benfenati F. Gasparini L. (2013). Lithium rescues synaptic plasticity and memory in Down syndrome mice. J. Clin. Invest. 123, 348–361. 10.1172/JCI64650 23202733 PMC3533293

[B26] Corrales A. Martínez P. García S. Vidal V. García E. Flórez J.. (2013). Long-term oral administration of melatonin improves spatial learning and memory and protects against cholinergic degeneration in middle-aged Ts65Dn mice, a model of Down syndrome. J. Pineal Res. 54, 346–358. 10.1111/jpi.12037 23350971

[B27] Corrales A. Vidal R. García S. Vidal V. Martínez P. García E.. (2014). Chronic melatonin treatment rescues electrophysiological and neuromorphological deficits in a mouse model of Down syndrome. J. Pineal Res. 56, 51–61. 10.1111/jpi.12097 24147912

[B28] Costa A. C. Grybko M. J. (2005). Deficits in hippocampal CA1 LTP induced by TBS but not HFS in the Ts65Dn mouse: a model of Down syndrome. Neurosci. Lett. 382, 317–322. 10.1016/j.neulet.2005.03.031 15925111

[B29] Costa A. C. Scott-McKean J. J. (2013). Prospects for improving brain function in individuals with down syndrome. CNS Drugs 27, 679–702. 10.1007/s40263-013-0089-3 23821040

[B30] Costa A. C. Scott-McKean J. J. Stasko M. R. (2008). Acute injections of the NMDA receptor antagonist memantine rescue performance deficits of the Ts65Dn mouse model of Down syndrome on a fear conditioning test. Neuropsychopharmacology 33, 1624–1632. 10.1038/sj.npp.1301535 17700645

[B31] Dang V. Medina B. Das D. Moghadam S. Martin K. J. Lin B.. (2014). Formoterol, a long-acting beta2 adrenergic agonist, improves cognitive function and promotes dendritic complexity in a mouse model of Down syndrome. Biol. Psychiatry. 75, 179–188. 10.1016/j.biopsych.2013.05.024 23827853

[B32] Das I. Park J. M. Shin J. H. Jeon S. K. Lorenzi H. Linden D. J.. (2013). Hedgehog agonist therapy corrects structural and cognitive deficits in a Down syndrome mouse model. Sci. Transl. Med. 5, 201ra120. 10.1126/scitranslmed.3005983 24005160 PMC4006719

[B33] Davisson M. T. Schmidt C. Akeson E. C. (1990). Segmental trisomy of murine chromosome 16: a new model system for studying Down syndrome. Prog. Clin. Biol. Res. 360, 263–280. 2147289

[B34] De la Torre R. De Sola S. Pons M. Duchon A. de Lagran M. M. Farré M.. (2014). Epigallocatechin-3-gallate, a DYRK1A inhibitor, rescues cognitive deficits in Down syndrome mouse models and in humans. Mol. Nutr. Food Res. 58, 278–288. 10.1002/mnfr.201300325 24039182

[B35] DeLong G. R. Ritch C. R. Burch S. (2002). Fluoxetine response in children with autistic spectrum disorders: correlation with familial major affective disorder and intellectual achievement. Dev. Med. Child Neurol. 44, 652–659. 10.1111/j.1469-8749.2002.tb00266.x 12418789

[B36] de Souza F. M. Busquet N. Blatner M. Maclean K. N. Restrepo D. (2011). Galantamine improves olfactory learning in the Ts65Dn mouse model of Down syndrome. Sci. Rep. 1:137. 10.1038/srep00137 22355654 PMC3216618

[B37] Dierssen M. (2012). Down syndrome: the brain in trisomic mode. Nat. Rev. Neurosci. 13, 844–858. 10.1038/nrn3314 23165261

[B38] Einarson A. Choi J. Einarson T. R. Koren G. (2009). Incidence of major malformations in infants following antidepressant exposure in pregnancy: results of a large prospective cohort study. Can. J. Psychiatry. 54, 242–246. 10.1016/j.ntt.2008.03.044 19321030

[B39] Eriksson P. S. Perfilieva E. Björk-Eriksson T. Alborn A. M. Nordborg C. Peterson D. A.. (1998). Neurogenesis in the adult human hippocampus. Nat. Med. 4, 1313–1317. 10.1038/3305 9809557

[B40] Faber K. M. Haring J. H. (1999). Synaptogenesis in the postnatal rat fascia dentata is influenced by 5-HT1a receptor activation. Brain Res. Dev. Brain Res. 114, 245–252. 10.1016/S0165-3806(99)00036-X 10320763

[B41] Faizi M. Bader P. L. Tun C. Encarnacion A. Kleschevnikov A. Belichenko P.. (2011). Comprehensive behavioral phenotyping of Ts65Dn mouse model of Down syndrome: activation of beta1-adrenergic receptor by xamoterol as a potential cognitive enhancer. Neurobiol. Dis. 43, 397–413. 10.1016/j.nbd.2011.04.011 21527343 PMC3539757

[B42] Fernandez F. Morishita W. Zuniga E. Nguyen J. Blank M. Malenka R. C.. (2007). Pharmacotherapy for cognitive impairment in a mouse model of Down syndrome. Nat. Neurosci. 10, 411–413. 10.1038/nn1860 17322876

[B43] Fortress A. M. Hamlett E. D. Vazey E. M. Aston-Jones G. Cass W. A. Boger H. A.. (2015). Designer receptors enhance memory in a mouse model of Down syndrome. J. Neurosci. 35, 1343–1353. 10.1523/JNEUROSCI.2658-14.2015 25632113 PMC4308587

[B44] Furu K. Kieler H. Haglund B. Selmer R. Stephannson O. Nǿrgaaed M. (2015). Selective serotonin reuptake inhibitors and venlafaxine in early pregnancy and risk of birth defects: population based cohort study and sibling design. BMJ 350:h1798. 10.1136/bmj.h1798 25888213 PMC4410618

[B45] Gardiner K. J. (2015). Pharmacological approaches to improving cognitive function in Down syndrome: current status and considerations. Drug Des. Devel. Ther. 9, 103–125. 10.2147/DDDT.S51476 25552901 PMC4277121

[B46] Gozes I. Divinski I. Piltzer I. (2008). NAP and D-SAL: neuroprotection against the beta amyloid peptide (1-42). BMC Neurosci. 9(Suppl. 3):S3. 10.1186/1471-2202-9-S3-S3 19091000 PMC2604881

[B47] Gozes I. Morimoto B. H. Tiong J. Fox A. Sutherland K. Dangoor D.. (2005). NAP: research and development of a peptide derived from activity-dependent neuroprotective protein (ADNP). CNS Drug Rev. 11, 353–368. 10.1111/j.1527-3458.2005.tb00053.x 16614735 PMC6741706

[B48] Granholm A. C. Ford K. A. Hyde L. A. Bimonte H. A. Hunter C. L. Nelson M.. (2002). Estrogen restores cognition and cholinergic phenotype in an animal model of Down syndrome. Physiol. Behav. 77, 371–385. 10.1016/S0031-9384(02)00884-3 12419414

[B49] Granholm A. C. Sanders L. Seo H. Lin L. Ford K. Isacson O. (2003). Estrogen alters amyloid precursor protein as well as dendritic and cholinergic markers in a mouse model of Down syndrome. Hippocampus 13, 905–914. 10.1002/hipo.10130 14750653

[B50] Guedj F. Bianchi D. W. (2013). Noninvasive prenatal testing creates an opportunity for antenatal treatment of Down syndrome. Prenat. Diagn. 33, 614–618. 10.1002/pd.4134 23595836

[B51] Guedj F. Bianchi D. W. Delabar J. M. (2014). Prenatal treatment of Down syndrome: a reality? Curr. Opin. Obstet. Gynecol. 26, 92–103. 10.1097/gco.0000000000000056 24573065

[B52] Guedj F. Sébrié C. Rivals I. Ledru A. Paly E. Bizot J. C.. (2009). Green tea polyphenols rescue of brain defects induced by overexpression of DYRK1A. PLoS ONE 4:e4606. 10.1371/journal.pone.0004606 19242551 PMC2645681

[B53] Guidi S. Bonasoni P. Ceccarelli C. Santini D. Gualtieri F. Ciani E.. (2008). Neurogenesis impairment and increased cell death reduce total neuron number in the hippocampal region of fetuses with Down syndrome. Brain Pathol. 18, 180–197. 10.1111/j.1750-3639.2007.00113.x 18093248 PMC8095525

[B54] Guidi S. Ciani E. Bonasoni P. Santini D. Bartesaghi R. (2011). Widespread proliferation impairment and hypocellularity in the cerebellum of fetuses with Down syndrome. Brain Pathol. 21, 361–373. 10.1111/j.1750-3639.2010.00459.x 21040072 PMC8094247

[B55] Guidi S. Stagni F. Bianchi P. Ciani E. Giacomini A. De Franceschi M.. (2014). Prenatal pharmacotherapy rescues brain development in a Down's syndrome mouse model. Brain 137, 380–401. 10.1093/brain/awt340 24334313

[B56] Guidi S. Stagni F. Bianchi P. Ciani E. Ragazzi E. Trazzi S.. (2013). Early pharmacotherapy with fluoxetine rescues dendritic pathology in the Ts65Dn mouse model of Down syndrome. Brain Pathol. 23, 129–143. 10.1111/j.1750-3639.2012.00624.x 22817700 PMC8028975

[B57] Gutierrez-Castellanos N. Winkelman B. H. Tolosa-Rodriguez L. Devenney B. Reeves R. H. De Zeeuw C. I. (2013). Size does not always matter: Ts65Dn Down syndrome mice show cerebellum-dependent motor learning deficits that cannot be rescued by postnatal SAG treatment. J. Neurosci. 33, 15408–15413. 10.1523/JNEUROSCI.2198-13.2013 24068809 PMC3858639

[B58] Hanson J. E. Weber M. Meilandt W. J. Wu T. Luu T. Deng L.. (2013). GluN2B antagonism affects interneurons and leads to immediate and persistent changes in synaptic plasticity, oscillations, and behavior. Neuropsychopharmacology 38, 1221–1233. 10.1038/npp.2013.19 23340518 PMC3656364

[B59] Hayes R. M. Wu P. Shelton R. C. Cooper W. O. Dupont W. D. Mitchel E.. (2012). Maternal antidepressant use and adverse outcomes: a cohort study of 228,876 pregnancies. Am. J. Obstet. Gynecol. 207, e41–e49. 10.1016/j.ajog.2012.04.028 22727349 PMC3567615

[B60] Heinen M. Hettich M. M. Ryan D. P. Schnell S. Paesler K. Ehninger D. (2012). Adult-onset fluoxetine treatment does not improve behavioral impairments and may have adverse effects on the Ts65Dn mouse model of Down syndrome. Neural Plast. 2012:467251. 10.1155/2012/467251 22848851 PMC3405721

[B61] Hollander E. Phillips A. Chaplin W. Zagursky K. Novotny S. Wasserman S.. (2005). A placebo controlled crossover trial of liquid fluoxetine on repetitive behaviors in childhood and adolescent autism. Neuropsychopharmacology 30, 582–589. 10.1038/sj.npp.1300627 15602505

[B62] Hunter C. L. Bachman D. Granholm A. C. (2004). Minocycline prevents cholinergic loss in a mouse model of Down's syndrome. Ann. Neurol. 56, 675–688. 10.1002/ana.20250 15468085

[B63] Imayoshi I. Sakamoto M. Ohtsuka T. Takao K. Miyakawa T. Yamaguchi M.. (2008). Roles of continuous neurogenesis in the structural and functional integrity of the adult forebrain. Nat. Neurosci. 11, 1153–1161. 10.1038/nn.2185 18758458

[B64] Incerti M. Horowitz K. Roberson R. Abebe D. Toso L. Caballero M.. (2012). Prenatal treatment prevents learning deficit in Down syndrome model. PLoS ONE 7:e50724. 10.1371/journal.pone.0050724 23209818 PMC3510191

[B65] Incerti M. Toso L. Vink J. Roberson R. Nold C. Abebe D.. (2011). Prevention of learning deficit in a Down syndrome model. Obstet. Gynecol. 117, 354–361. 10.1097/AOG.0b013e3182051ca5 21252750

[B66] Isbrucker R. A. Edwards J. A. Wolz E. Davidovich A. Bausch J. (2006). Safety studies on epigallocatechin gallate (EGCG) preparations. Part 3: teratogenicity and reproductive toxicity studies in rats. Food Chem. Toxicol. 55, 651–661. 10.1016/j.fct.2005.11.002 16410036

[B67] Kelley C. M. Powers B. E. Velazquez R. Ash J. A. Ginsberg S. D. Strupp B. J.. (2014). Maternal choline supplementation differentially alters the basal forebrain cholinergic system of young-adult Ts65Dn and disomic mice. J. Comp. Neurol. 522, 1390–1410. 10.1002/cne.23492 24178831 PMC3959592

[B68] Kim H. S. Quon M. J. Kim J. A. (2014). New insights into the mechanisms of polyphenols beyond antioxidant properties; lessons from the green tea polyphenol, epigallocatechin 3-gallate. Redox Biol. 2, 187–195. 10.1016/j.redox.2013.12.022 24494192 PMC3909779

[B69] Kleschevnikov A. M. Belichenko P. V. Faizi M. Jacobs L. F. Htun K. Shamloo M.. (2012). Deficits in cognition and synaptic plasticity in a mouse model of Down syndrome ameliorated by GABAB receptor antagonists. J. Neurosci. 32, 9217–9227. 10.1523/JNEUROSCI.1673-12.2012 22764230 PMC3411326

[B70] Kleschevnikov A. M. Belichenko P. V. Villar A. J. Epstein C. J. Malenka R. C. Mobley W. C. (2004). Hippocampal long-term potentiation suppressed by increased inhibition in the Ts65Dn mouse, a genetic model of Down syndrome. J. Neurosci. 24, 8153–8160. 10.1523/JNEUROSCI.1766-04.2004 15371516 PMC6729789

[B71] Lambert J. D. Kennett M. J. Sang S. Rehul K. R. Ju J. Yang C. S. (2009). Hepatotoxicity of High Oral Dose (-)-Epigallocatechin-3-Gallate in Mice. Food Chem. Toxicol. 2010, 409–416. 10.1016/j.fct.2009.10.030 19883714 PMC2905152

[B72] Latchney S. E. Jaramillo T. C. Rivera P. D. Eisch A. J. Powell C. M. (2015). Chronic P7C3 treatment restores hippocampal neurogenesis. Neurosci. Lett. 591, 86–92. 10.1016/j.neulet.2015.02.008 25668489 PMC4363293

[B73] Lockrow J. Boger H. Bimonte-Nelson H. Granholm A. C. (2011). Effects of long-term memantine on memory and neuropathology in Ts65Dn mice, a model for Down syndrome. Behav. Brain Res. 221, 610–622. 10.1016/j.bbr.2010.03.036 20363261 PMC2928411

[B74] Lockrow J. Prakasam A. Huang P. Bimonte-Nelson H. Sambamurti K. Granholm A. C. (2009). Cholinergic degeneration and memory loss delayed by vitamin E in a Down syndrome mouse model. Exp. Neurol. 216, 278–289. 10.1016/j.expneurol.2008.11.021 19135442 PMC2704550

[B75] Lysenko L. V. Kim J. Henry C. Tyrtyshnaia A. Kohnz R. A. Madamba F.. (2014). Monoacylglycerol lipase inhibitor JZL184 improves behavior and neural properties in Ts65Dn mice, a model of down syndrome. PLoS ONE 9:e114521. 10.1371/journal.pone.0114521 25474204 PMC4256450

[B76] Martínez-Cué C. Martínez P. Rueda N. Vidal R. García S. Vidal V.. (2013). Reducing GABAA alpha5 receptor-mediated inhibition rescues functional and neuromorphological deficits in a mouse model of down syndrome. J. Neurosci. 33, 3953–3966. 10.1523/JNEUROSCI.1203-12.2013 23447605 PMC6619314

[B77] Moon J. Chen M. Gandhy S. U. Strawderman M. Levitsky D. A. Maclean K. N.. (2010). Perinatal choline supplementation improves cognitive functioning and emotion regulation in the Ts65Dn mouse model of Down syndrome. Behav. Neurosci. 124, 346–361. 10.1037/a0019590 20528079 PMC2955960

[B78] Moran T. H. Capone G. T. Knipp S. Davisson M. T. Reeves R. H. Gearhart J. D. (2002). The effects of piracetam on cognitive performance in a mouse model of Down's syndrome. Physiol. Behav. 77, 403–409. 10.1016/S0031-9384(02)00873-9 12419416

[B79] Moses-Kolko E. L. Bogen D. Perel J. Bregar A. Uhl K. Levin B.. (2005). Neonatal signs after late *in utero* exposure to serotonin reuptake inhibitors: literature review and implications for clinical applications. JAMA 293, 2372–2383. 10.1001/jama.293.19.2372 15900008

[B80] Netzer W. J. Powell C. Nong Y. Blundell J. Wong L. Duff K.. (2010). Lowering beta-amyloid levels rescues learning and memory in a Down syndrome mouse model. PLoS ONE 5:e10943. 10.1371/journal.pone.0010943 20532168 PMC2880593

[B81] Olivier J. Akerud H. Kaihola L. Pawluski J. Skalkidou S. Högberg U.. (2013). The effects of maternal depression and maternal selective serotonin reuptake inhibitor exposure on offspring. Front. Cell Neurosci. 7:73. 10.3389/fncel.2013.00073 23734100 PMC3659337

[B82] Pedersen L. H. Henriksen T. B. Olsen J. (2010). Fetal exposure to antidepressants and normal milestone development at 6 and 19 months of age. Pediatrics 125, e600–e608. 10.1542/peds.2008-3655 20176667

[B83] Perrone S. Longini M. Bellieni C. V. Centini G. Kenanidis A. De Marco L.. (2007). Early oxidative stress in amniotic fluid of pregnancies with Down syndrome. Clin. Biochem. 40, 177–180. 10.1016/j.clinbiochem.2006.10.019 17208212

[B84] Pons-Espinal M. Martinez de Lagran M. Dierssen M. (2013). Environmental enrichment rescues DYRK1A activity and hippocampal adult neurogenesis in TgDyrk1A. Neurobiol. Dis. 60, 18–31. 10.1016/j.nbd.2013.08.008 23969234

[B85] Rachidi M. Lopes C. (2008). Mental retardation and associated neurological dysfunctions in Down syndrome: a consequence of dysregulation in critical chromosome 21 genes and associated molecular pathways. Eur. J. Paediatr. Neurol. 12, 168–182. 10.1016/j.ejpn.2007.08.010 17933568

[B86] Reefhuis J. Devine O. Friedman J. M. Louik K. Honein M. (2015). Specific SSRIs and birth defects: bayesian analysis to interpret new data in the context of previous reports. BMJ 350:h3190. 10.1136/bmj.h3190 26156519 PMC4496787

[B87] Rice D. Barone S. (2010). Critical periods of vulnerabiliy for the developing nervpus system: evidence from humans and animal models. Environ. Health Perspect. 108(Suppl. 3), 511–533. 10.1289/ehp.00108s3511 10852851 PMC1637807

[B88] Roper R. J. Baxter L. L. Saran N. G. Klinedinst D. K. Beachy P. A. Reeves R. H. (2006). Defective cerebellar response to mitogenic Hedgehog signaling in Down [corrected] syndrome mice. Proc. Natl. Acad. Sci. U.S.A. 103, 1452–1456. 10.1073/pnas.0510750103 16432181 PMC1360600

[B89] Rueda N. Florez J. Martínez-Cué C. (2008a). Chronic pentylenetetrazole but not donepezil treatment rescues spatial cognition in Ts65Dn mice, a model for Down syndrome. Neurosci. Lett. 433, 22–27. 10.1016/j.neulet.2007.12.039 18226451

[B90] Rueda N. Flórez J. Martínez-Cué C. (2008b). Effects of chronic administration of SGS-111 during adulthood and during the pre- and post-natal periods on the cognitive deficits of Ts65Dn mice, a model of Down syndrome. Behav. Brain Res. 188, 355–367. 10.1016/j.bbr.2007.11.020 18178265

[B91] Rueda N. Flórez J. Martínez-Cué C. (2012). Mouse models of Down syndrome as a tool to unravel the causes of mental disabilities. Neural Plast. 2012, 1–26. 10.1155/2012/584071 22685678 PMC3364589

[B92] Rueda N. Llorens-Martín M. Flórez J. Valdizán E. Banerjee P. Trejo J. L.. (2010). Memantine normalizes several phenotypic features in the Ts65Dn mouse model of Down syndrome. J. Alzheimers Dis. 21, 277–290. 10.3233/JAD-2010-100240 20421694

[B93] Salehi A. Faizi M. Colas D. Valletta J. Laguna J. Takimoto-Kimura R.. (2009). Restoration of norepinephrine-modulated contextual memory in a mouse model of Down syndrome. Sci. Transl. Med. 1, 7ra17. 10.1126/scitranslmed.3000258 20368182

[B94] Sanz E. J. De-las-Cuevas C. Kiuru A. Bate A. Edwards R. (2005). Selective serotonin reuptake inhibitors in pregnant women and neonatal withdrawal syndrome: a database analysis. Lancet 365, 482–487. 10.1016/S0140-6736(05)70271-3 15705457

[B95] Seress L. Abraham H. Tornóczky T. Kosztolányi G. (2001). Cell formation in the human hippocampal formation from mid-gestation to the late postnatal period. Neuroscience 105, 831–843. 10.1016/S0306-4522(01)00156-7 11530221

[B96] Shichiri M. Yoshida Y. Ishida N. Hagihara Y. Iwahashi H. Tamai H.. (2011). Alpha-Tocopherol suppresses lipid peroxidation and behavioral and cognitive impairments in the Ts65Dn mouse model of Down syndrome. Free Radic. Biol. Med. 50, 1801–1811. 10.1016/j.freeradbiomed.2011.03.023 21447382

[B97] Sillitoe R. V. Joyner A. L. (2007). Morphology, molecular codes, and circuitry produce the three-dimensional complexity of the cerebellum. Annu. Rev. Cell Dev. Biol. 23, 549–577. 10.1146/annurev.cellbio.23.090506.123237 17506688

[B98] Stagni F. Giacomini A. Guidi S. Ciani E. Ragazzi E. Filonzi M.. (2015). Long-term effects of neonatal treatment with fluoxetine on cognitive performance in Ts65Dn mice. Neurobiol. Dis. 74C, 204–218. 10.1016/j.nbd.2014.12.005 25497735

[B99] Stagni F. Magistretti J. Guidi S. Ciani E. Mangano C. Calzà L.. (2013). Pharmacotherapy with fluoxetine restores functional connectivity from the dentate gyrus to field CA3 in the Ts65Dn mouse model of Down syndrome. PLoS ONE 8:e61689. 10.1371/journal.pone.0061689 23620781 PMC3631158

[B100] Stagni F. Trazzi S. Giacomini A. Guidi S. Emili M. Ciani E. (2014). Treatment with Epigallocatechin Gallate rescues neurogenesis and neuron maturation in the Ts65Dn mouse model of Down syndrome. XXII National Congress of the Italian Society of Psychophysiology. 27th-29th November 2014. Neuopsychological Trends. 16, 120.

[B101] Stiles J. Jernigan T. L. (2010). The basics of brain development. Neuropsychol. Rev. 20, 327–348. 10.1007/s11065-010-9148-4 21042938 PMC2989000

[B102] Sudarov A. Joyner A. L. (2007). Cerebellum morphogenesis: the foliation pattern is orchestrated by multi-cellular anchoring centers. Neural Dev. 2:26. 10.1186/1749-8104-2-26 18053187 PMC2246128

[B103] Takahashi T. Nowakowski R. S. Caviness V. S. Jr. (1996). The leaving or Q fraction of the murine cerebral proliferative epithelium: a general model of neocortical neuronogenesis. J. Neurosci. 16, 6183–6196. 8815900 10.1523/JNEUROSCI.16-19-06183.1996 PMC6579174

[B104] Takashima S. Ieshima A. Nakamura H. Becker L. E. (1989). Dendrites, dementia and the Down syndrome. Brain Dev. 11, 131–133. 10.1016/S0387-7604(89)80082-8 2523670

[B105] ten Donkelaar H. J. Lammens M. Wesseling P. Thijssen H. O. Renier W. O. (2003). Development and developmental disorders of the human cerebellum. J. Neurol. 250, 1025–1036. 10.1007/s00415-003-0199-9 14504962

[B106] Toso L. Cameroni I. Roberson R. Abebe D. Bissell S. Spong C. Y. (2008). Prevention of developmental delays in a Down syndrome mouse model. Obstet. Gynecol. 112, 1242–1251. 10.1097/AOG.0b013e31818c91dc 19037032 PMC2687469

[B107] Trazzi S. Mitrugno V. M. Valli E. Fuchs C. Rizzi S. Guidi S.. (2011). APP-dependent up-regulation of Ptch1 underlies proliferation impairment of neural precursors in Down syndrome. Hum. Mol. Genet. 20, 1560–1573. 10.1093/hmg/ddr033 21266456

[B108] Vacca R. A. Valenti D. (2015). Green tea EGCG plus fish oil omega-3 dietary supplements rescue mitochondrial dysfunctions and are safe in a Down's syndrome child. Clin. Nutr. 34, 783–784. 10.1016/j.clnu.2015.04.012 25962746

[B109] Velazquez R. Ash J. A. Powers B. E. Kelley C. M. Strawderman M. Luscher Z. I.. (2013). Maternal choline supplementation improves spatial learning and adult hippocampal neurogenesis in the Ts65Dn mouse model of Down syndrome. Neurobiol. Dis. 58, 92–101. 10.1016/j.nbd.2013.04.016 23643842 PMC4029409

[B110] Vidal V. García S. Martínez P. Corrales A. Flórez J. Rueda N.. (2012). Lack of behavioral and cognitive effects of chronic ethosuximide and gabapentin treatment in the Ts65Dn mouse model of Down syndrome. Neuroscience 220, 158–168. 10.1016/j.neuroscience.2012.06.031 22728103

[B111] Vink J. Incerti M. Toso L. Roberson R. Abebe D. Spong C. Y. (2009). Prenatal NAP+SAL prevents developmental delay in a mouse model of Down syndrome through effects on N-methyl-D-aspartic acid and gamma-aminobutyric acid receptors. Am. J. Obstet. Gynecol. 200, e521–e524. 10.1016/j.ajog.2009.01.052 19327737 PMC6503529

[B112] Whitaker-Azmitia P. M. (2001). Serotonin and brain development: role in human developmental diseases. Brain Res. Bull. 56, 479–485. 10.1016/S0361-9230(01)00615-3 11750793

[B113] Xie W. Ramakrishna N. Wieraszko A. Hwang Y. W. (2008). Promotion of neuronal plasticity by (-)-epigallocatechin-3-gallate. Neurochem. Res. 33, 776–783. 10.1007/s11064-007-9494-7 17943438

[B114] Yan Y. Jiang X. West A. A. Perry C. A. Malysheva O. V. Brenna J. T.. (2013). Pregnancy alters choline dynamics: results of a randomized trial using stable isotope methodology in pregnant and nonpregnant women. Am. J. Clin. Nutr. 98, 1459–1467. 10.3945/ajcn.113.066092 24132975 PMC6410899

